# Crystal structure and theoretical studies of two π-conjugated fused-ring chalcones: (*E*)-1-(anthra­cen-9-yl)-3-(9-ethyl-9*H*-carbazol-3-yl)prop-2-en-1-one and (*E*)-1-(anthracen-9-yl)-3-[4-(9*H*-carbazol-9-yl)phen­yl]prop-2-en-1-one

**DOI:** 10.1107/S2056989018011131

**Published:** 2018-08-21

**Authors:** Dian Alwani Zainuri, Ibrahim Abdul Razak, Suhana Arshad

**Affiliations:** a X-ray Crystallography Unit, School of Physics, Universiti Sains Malaysia, 11800 USM, Penang, Malaysia

**Keywords:** chalcone, anthracene, crystal structure, DFT

## Abstract

The title chalcones, C_31_H_23_NO and C_35_H_23_NO, were synthesized *via* Claisen–Schmidt condensation reactions. Both structures were solved and refined using single-crystal X-ray diffraction data and optimized at the ground state using the density functional theory (DFT) method with the B3LYP/6-311++G(d,p) level. In the crystals, π–π inter­ations and weak C—H⋯O and C—H⋯π inter­actions are observed. The effect of these inter­molecular inter­actions in the solid state can be seen by the difference between the experimental and theoretical optimized geometrical parameters. The structures have also been characterized by UV–Vis spectroscopy. The smallest energy gaps of 2.86 and 2.96 eV enhance the nonlinear responses of such mol­ecular systems. Hirshfeld surface analyses and 2D (two-dimensional) fingerprint plots were used to qu­antify the inter­molecular inter­actions present in the crystal, indicating that these are the most important contribution to the crystal packing.

## Abstract 2

Two chalcones were synthesized in Claisen–Schmidt condensation reactions. In the crystals, π–π inter­actions and weak C—H⋯O and C—H⋯π inter­actions are observed. The effect of these inter­molecular inter­actions in the solid state can be seen inthe difference between the experimental and theoretical optimized geometrical parameters.

## Chemical context   

Chalcones satisfy the criteria of three features essential for high nonlinear activity in an organic compound, which are: a strong electron donor, a highly polarizable π-conjugated bridge and a strong π-electron acceptor. A chalcone mol­ecule with a π-conjugated system provides a large charge-transfer axis with appropriate substituent groups on the terminal aromatic rings. Polyaromatic hydro­carbons or π-conjugated materials such as anthracenyl chalcone provide the significant property for conductivity that led to tremendous advances in the field of organic electronics (Li *et al.*, 2016 ▸). These conjugated materials modifications on the anthracenyl chalcone decrease the HOMO–LUMO energy gap (HOMO is the highest occupied molecular orbital and LUMO is the lowest unoccupied mol­ecular orbital), enhancing the nonlinear responses of such mol­ecular systems. In this work, we report the synthesis and combined experimental and theoretical studies of the anthracene chalcones (*E*)-1-(anthracen-9-yl)-3-(9-ethyl-9*H*-carbazol-3-yl)prop-2-en-1-one, **I**, and (*E*)-1-(anthracen-9-yl)-3-[4-(9*H*-carbazol-9-yl)phen­yl]prop-2-en-1-one, **II**. Additionally, the UV–vis absorption and Hirshfeld surface analyses are discussed.

## Structural commentary   

**Figure 1:**
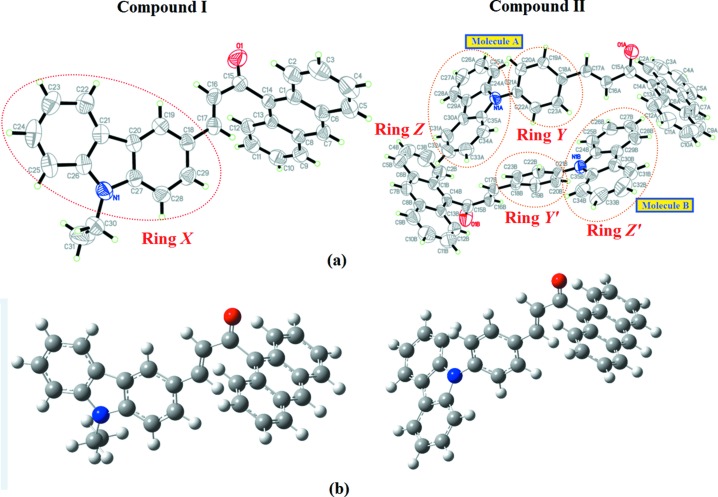
(*a*) The mol­ecular structures of compounds **I** and **II**. (*b*) The optimized structures of compounds **I** and **II** at DFT/B3LYP 6-311++G(d,p).

The mol­ecular structures and optimized geometries of compounds **I** and **II** are shown in Fig. 1 ▸. The optimization of the mol­ecular geometries leading to energy minima was achieved using DFT with a 6-311++G(d,p) basis set, as implemented in the *GAUSSIAN09* program package (Frisch *et al.*, 2009 ▸). The calculated geometric parameters, such as bond lengths, bond angles and torsion angles, compared to the experimental data are presented in Table S1 in the supporting information and exhibit normal ranges. The theoretical bond lengths, bond angles and torsion angles correlate well with the experimental data.
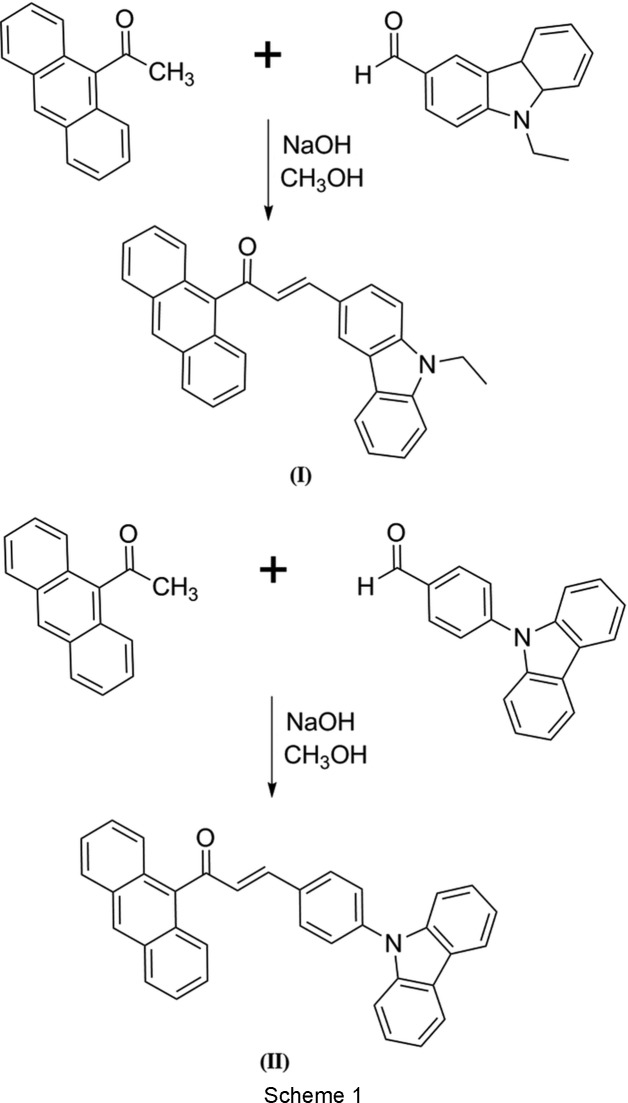


Both **I** and **II** comprise a chalcone with an anthracene ring with 9-ethyl-9*H*-carbazole and 9-phenyl-9*H*-carbazole substituents, respectively. The asymmetric unit of **II** contains two crystallographically independent mol­ecules, **A** and **B** (Fig. 1 ▸ *a*). The C—C distances in the central ring of the anthracene units show a little variations compared to the other rings (C2—C3, C4—C5, C9—C10 and C11—C12), which are much shorter. These observations are consistent with an electronic structure for the anthracene units where a central ring displaying aromatic delocalization is flanked by two isolated diene units (Glidewell & Lloyd, 1984 ▸).

Both theoretical and experimental structures (Fig. 1 ▸) exist in an *s-trans* configuration with respect to the enone moiety, with bond lengths C15=O1 [Exp = 1.220 (2) Å and DFT = 1.22 Å in **I**; Exp = 1.213 (3) (**A**) and 1.218 (3) Å (**B**), and DFT = 1.22 Å in **II**] and C16=C17 [Exp = 1.329 (2) Å and DFT = 1.35 Å in **I**; Exp = 1.319 (3) (**A**) and 1.320 (4) Å (**B**), and DFT = 1.35 Å in **II**]. Both **I** and **II** (**A** and **B**) are twisted at the C14—C15 bond, with C1—C14—C15—C16 torsion angles of −92.6 (2) (in **I**), 84.8 (3) (in **IIA**) and 106.3 (3)° (in **IIB**). The corresponding torsion angles for DFT are −85.84 and 85.63°, respectively. Additionally, in compound **II**, rings *Y* and *Z* (**A**) and rings *Y′* and *Z′* (**B**) are also twisted at the C21—N1 bond, with C20—C21—N1—C24 torsion angles of Exp = 64.1 (4)° (**A**) and 46.2 (4)° (**B**), and DFT = 55.03°. The large twist angles are due to the bulkiness of the strong electron-donor anthracene ring system and substituent ring system (Zainuri *et al.*, 2018*a* ▸,*b* ▸,*c* ▸). Meanwhile, compounds **I** and **II** are found to be slightly twisted at the C17—C18 bond, with C16—C17—C18—C19 torsion angles of Exp = −16.4 (3)° and DFT = −1.38 for compound **I**, and Exp = −171.2 (3)° (**A**) and 11.4 (5)° (**B**), and DFT = −1.70° for compound **II**. The slight differences in the torsion angles between the experimental and DFT results in both compounds are due to the formation of inter­molecular C—H⋯O and C—H⋯π inter­actions involving all the fused-ring systems, which are not taken into consideration during the optimization process (Arshad *et al.*, 2018 ▸).

The enone moiety in **I** [O1/C15–C17, maximum deviation = 0.0308 (19) Å at atom C16] makes dihedral angles of 86.93 (19) and 21.21 (19)° with the anthracene ring [maximum deviation = 0.0117 (19) Å at C9] and ring *X* [maximum deviation = 0.0363 (18) Å at C29], respectively. In compound **II**, the enone moiety [O1/C15–C17, maximum deviation = 0.017 (3) Å at C15*A*] for mol­ecule **A** forms dihedral angles of 84.76 (17), 87.61 (17) and 72.35 (17)° with the anthracene ring [maximum deviation = 0.029 (3) Å at C14*A*], ring *Y* [maximum deviation = 0.008 (3) Å at C19*A*] and ring *Z* [maximum deviation = 0.043 (3) Å at C34*A*], respectively. The anthracene ring forms dihedral angles of 89.63 (11) and 62.11 (7)° with rings *Y* and *Z*, respectively, and the dihedral angle between rings *Y* and *Z* is 61.73 (10)°. In addition, for mol­ecule **B**, the enone moiety [O1/C15–C17, maximum deviation = 0.036 (3) Å at C16*B*] forms dihedral angles of 72.2 (3), 13.5 (3) and 87.2 (3)° with the anthracene ring [maximum deviation = 0.018 (4) Å at C10*B*], ring *Y′* [maximum deviation = 0.010 (3) Å at C20*B*] and ring *Z′* [maximum deviation = 1.441 (2) Å at N1*B*], respectively. The anthracene ring forms dihedral angles of 61.46 (11) and 54.80 (7)° with rings *Y′* and *Z′*, respectively, and the dihedral angle between rings *Y′* and *Z′* is 48.92 (11)°.

## Supra­molecular features   

**Figure 2:**
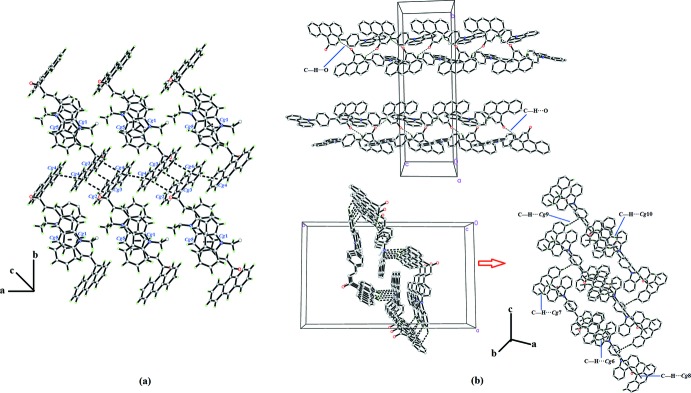
The crystal packing showing (*a*) weak π–π inter­actions in compound **I** and (*b*) weak C—H⋯O and C—H⋯π inter­actions of compound **II**.

The crystal packing of **I** shows weak π–π inter­actions (Fig. 2 ▸ *a*) involving *Cg*1⋯*Cg*5 = 3.7267 (11) Å (symmetry code: 1 − *x*, 1 − *y*, 1 − *z*), *Cg*2⋯*Cg*4 = 3.6669 (12) Å (symmetry code: 2 − *x*, 2 − *y*, 1 − *z*), *Cg*3⋯*Cg*3 = 3.6585 (11) Å (symmetry code: 2 − *x*, 2 − *y*, 1 − *z*) and *Cg*4⋯*Cg*4 = 3.6790 (12) Å (symmetry code: 1 − *x*, 2 − *y*, 1 − *z*), where *Cg*1, *Cg*2, *Cg*3, *Cg*4 and *Cg*5 are the centroids of rings N1/C20/C21/C26/C27, C1–C6, C1/C6–C8/C13/C14, C8–C13, C18–C20/C27-C29, respectively. The packing is further linked into an infinite three-dimensional supra­molecular network.

Lists of weak hydrogen-bond inter­molecular inter­actions are shown in Table 1 ▸. The crystal packing of **II** (Fig. 1 ▸ *b*) shows weak C12*B*—H12*B*⋯O1 inter­molecular hydrogen bonds connecting the mol­ecules into an infinite one-dimensional chain along the *c* axis. In addition, weak inter­molecular C5*B*—H5*BA*⋯*Cg*6, C27*B*—H27*B*⋯*Cg*7, C28*B*—H28*B*⋯*Cg*8, C11*A*—H11*A*⋯*Cg*9 and C7*B*—H7*B*⋯*Cg*10 inter­actions are also observed in the crystal packing and further stabilize the crystal structure, where *Cg*6, *Cg*7, *Cg*8, *Cg*9 and *Cg*10 are the centroids of rings N1*A*/C24*A*/C29*A*/C30*A*/C35*A*, C1*A*–C6*A*, C1*A*/C6*A*–C8*A*/C13*A*/C14*A*, C18*A*–C23*A* and C24*A*–C29*A*, respectively. These weak inter­molecular C—H⋯O and C—H⋯π inter­actions bridge the mol­ecules into an infinite one-dimensional column along the *c* axis.

## UV–Vis absorption analysis   

**Figure 3:**
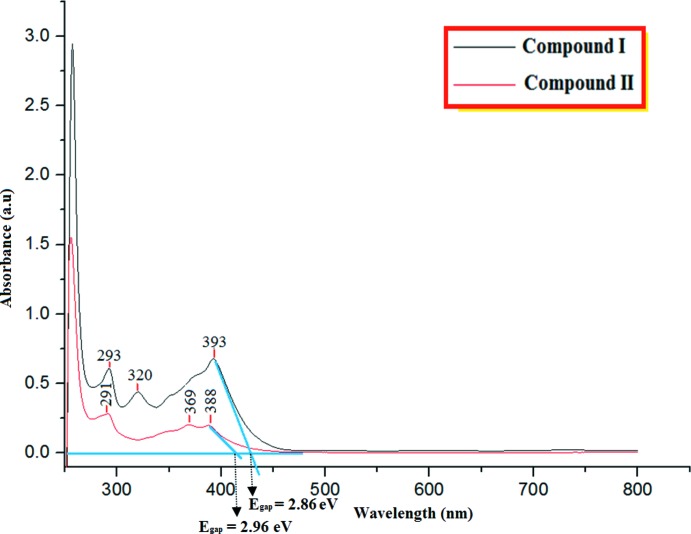
The UV–Vis absorption spectra of compounds **I** and **II**.

The electronic absorption spectra of **I** and **II** have been calculated using time-dependent DFT at the B3LYP/6-311++G(d,p) level in the gas phase and give values of 396 (**I**) and 383 nm (**II**). The absorption characteristics of **I** and **II** are observed in the UV region at 393 and 388 nm, as shown in Fig. 3 ▸. The theoretical wavelengths are shifted to higher values and are due to the fact that the calculations are confined to the gaseous medium, whereas the observations are from the solution state, using DMSO as solvent (Zainuri *et al.*, 2017 ▸).

**Figure 4:**
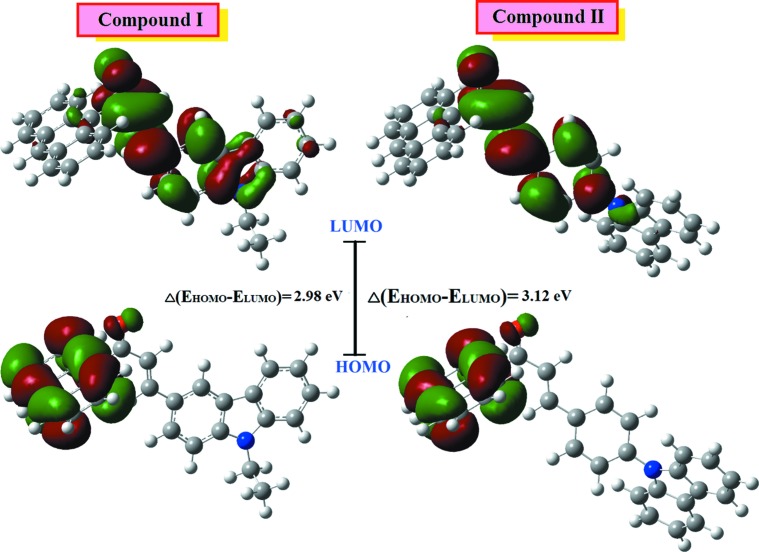
The electron distribution of the HOMO and LUMO energy levels of compounds **I** and **II**.

According to an investigation on the frontier mol­ecular orbital (FMO) energy levels of the title compounds, the corresponding electronic transfer are found to happen between the HOMO and LUMO orbitals, as shown in Fig. 4 ▸. The positive phase is red and the negative is green. In Fig. 4 ▸, the charge densities in the ground state (HOMO) are mainly delocalized over the anthracenyl donor ring, while in the excited state (LUMO), the charge densities were accumulated on the π-conjugated enone bridge and the terminal electron acceptor group. The values of the energy separations between the HOMO and LUMO are 2.98 and 3.12 eV for compounds **I** and **II**, respectively. Through an extrapolation of the linear trend observed in the optical spectra, the experimental energy band gaps in **I** and **II** are 2.86 and 2.96 eV, respectively. These optical band-gap values indicate the suitability of this compound for optoelectronic applications, as was also reported previously for a chalcone structure by Tejkiran *et al.* (2016 ▸). In addition, Konkol *et al.* (2016 ▸) studied the structural and optical properties of fused rings where the results showed that fused rings have a lower energy band gap.

## Hirshfeld surface (HS) analysis   

**Figure 5:**
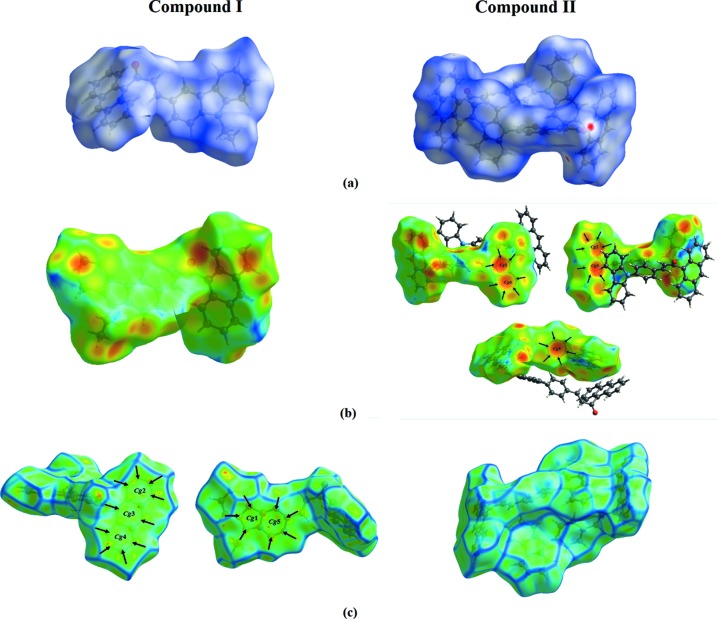
View of the Hirshfeld surfaces for the title compounds, showing (*a*) *d*_norm_ with the red spots showing the involvement of the C—H⋯O inter­actions in **II**, (*b*) mapped over *d*_e_ with the pale-orange spots within the black arrows indicating the C—H⋯π inter­actions in **II** and (*c*) mapped over curvedness with the black arrows indicating the π–π inter­actions in **I**.

The program *CrystalExplorer* (Wolff *et al.*, 2012 ▸) was used to analyse the inter­actions in the crystal. Fig. 5 ▸(*a*) show the HS mapped over *d*_norm_, where the red spots indicate the regions of donor–acceptor inter­actions. The C—H⋯O contacts are only present in compound **II**. In addition, the presence of C—H⋯π inter­actions only occurs in compound **II**, indicated through a combination of pale-orange bright-red spots which are present on the HS mapped over shape index surface, identified with black arrows (Fig. 5 ▸ *b*). The large flat region delineated by a blue outline refers to the π–π stacking inter­actions. The curved nature of the compound reveals that π–π stacking inter­actions are present in compound **I**. Meanwhile, these inter­actions are absent in compound **II**.

**Figure 6:**
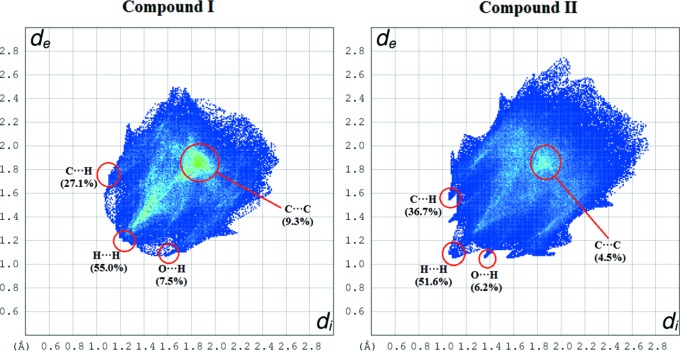
Fingerprint plots of the H⋯H, H⋯O, C⋯H and C⋯C inter­actions showing the relative contributions to the total Hirshfeld surface.

The fingerprint plot shown in Fig. 6 ▸ indicates the H⋯H, H⋯O, C⋯H and C⋯C inter­actions with their relative percentage contributions. The H⋯H contacts have the largest overall contribution to the HS, and these inter­actions dominate in the crystal structure. The contribution from H⋯O/O⋯H contacts to the HS showing two narrow spikes provides evidence for the presence of inter­molecular C—H⋯O inter­actions in Fig. 6 ▸ for compound **II**. Meanwhile, there is no spike in the fingerprint of compound **I**. The 7.5% O⋯H contribution shown in compound **I** is the average percentage interaction from the total interactions presence in **I**. In compound **I**, there are no interactions other than the π–π interactions, which makes the percentage of the O⋯H contribution is slightly higher. Hence, a discussion on the percentage difference between **I**. and **II**. is invalid. The significant C—H⋯π inter­actions for compound **II** are indicated by the wings *d*_e_ + *d*_i_ ∼ 2.6 Å.

## Database survey   

A survey of the Cambridge Structural Database (CSD, Version 5.39, last update November 2017; Groom *et al.*, 2016 ▸) revealed several fused-ring-substituted chalcones similar to **I** and **II**. There are four compounds which have an anthrancene ketone subtituent on the chalcone, including 9-anthryl styryl ketone and 9,10-anthryl bis­(styryl ketone) reported by Harlow *et al.* (1975 ▸). (2*E*)-1-(Anthracen-9-yl)-3-[4-(propan-2-yl)phen­yl]prop-2-en-1-one was reported by Girisha *et al.* (2016 ▸), while (*E*)-1-(anthracen-9-yl)-3-(2-chloro-6-fluoro­phen­yl)prop-2-en-1-one was reported by Abdullah *et al.* (2016 ▸). Zainuri *et al.* (2018*a* ▸) reported both anthrancene substituents on chalcone (*E*)-1,3-bis­(anthracen-9-yl)prop-2-en-1-one. Other related compounds include 1-(anthracen-9-yl)-2-methyl­prop-2-en-1-one (Agrahari *et al.*, 2015 ▸), 9-anthroylacetone (Cicogna *et al.*, 2004 ▸), (*E*)-1-(anthracen-9-yl)-3-[4-(piperidin-1-yl)phen­yl]prop-2-en-1-one and (*E*)-1-(anthracen-9-yl)-3-[4-(di­phenyl­amino)­phen­yl]prop-2-en-1-one (Zainuri *et al.*, 2018*b* ▸,*c* ▸).

## Synthesis and crystallization   

A mixture of 9-acetyl­anthrancene (0.5 mmol) and 9-ethylcarbazole-3-carbaldehyde (0.5 mmol) and 4-(9*H*-carbazol-9-yl)benzaldehyde (0.5 mmol) for compounds **I** and **II**, respectively, was dissolved in methanol (20 ml). A catalytic amount of NaOH (5 ml, 20%) was added to the solution dropwise under vigorous stirring. The reaction mixture was stirred for about 5–6 h at room temperature. After stirring, the contents of the flask were poured into ice-cold water (50 ml). The resultant crude products were filtered, washed successively with distilled water and recrystallized from acetone to give the corresponding chalcones (Scheme 1). Single crystals of **I** and **II** suitable for X-ray diffraction were obtained by slow evaporation from acetone solutions.

## Refinement   

Crystal data collection and structure refinement details are summarized in Table 2 ▸. All H atoms were positioned geometrically (C—H = 0.93, 0.96 and 0.97 Å in **I**, and 0.93 Å in **II**) and refined using a riding model, with *U*_iso_(H) = 1.2 or 1.5*U*_eq_(C). A rotating group model was applied to the methyl group in **I**.

## Supplementary Material

Crystal structure: contains datablock(s) mo_DA20_0m, mo_DA21e_0m, global. DOI: 10.1107/S2056989018011131/lh5878sup1.cif

Structure factors: contains datablock(s) mo_DA20_0m. DOI: 10.1107/S2056989018011131/lh5878mo_DA20_0msup2.hkl

Supporting information file. DOI: 10.1107/S2056989018011131/lh5878mo_DA20_0msup4.cml

Structure factors: contains datablock(s) mo_DA21e_0m. DOI: 10.1107/S2056989018011131/lh5878mo_DA21e_0msup3.hkl

Supporting information file. DOI: 10.1107/S2056989018011131/lh5878sup5.pdf

CCDC references: 1827021, 1827019

Additional supporting information:  crystallographic information; 3D view; checkCIF report

## Figures and Tables

**Table 1:** Hydrogen-bond geometry (Å, °) for **II** [Chem scheme1]

*D*—H⋯*A*	*D*—H	H⋯*A*	*D*⋯*A*	*D*—H⋯*A*
C12*B*—H12*B*⋯O1*B* ^i^	0.93	2.51	3.266 (4)	138
C5*B*—H5*BA*⋯*Cg*6^ii^	0.93	2.79	3.585 (4)	144
C27*B*—H27*B*⋯*Cg*7	0.93	2.85	3.577 (4)	136
C28*B*—H28*B*⋯*Cg*8	0.93	2.70	3.382 (4)	130
C11*A*—H11*A*⋯*Cg*9^iii^	0.93	2.85	3.742 (4)	161
C7*B*—H7*BA*⋯*Cg*10^ii^	0.93	2.90	3.704 (3)	145

Symmetry codes: (i) 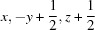; (ii) 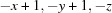; (iii) 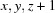.

**Table 2:** Experimental details

	**I**	**II**
Crystal data		
Chemical formula	C_31_H_23_NO	C_35_H_23_NO
*M*_r_	425.50	473.54
Crystal system, space group	Monoclinic, *P*2_1_/*c*	Monoclinic, *P*2_1_/*c*
Temperature (K)	296	296
*a*, *b*, *c* (Å)	9.3038 (11), 15.0166 (18), 16.1170 (19)	18.019 (3), 29.214 (4), 9.5503 (13)
β (°)	99.286 (2)	97.637 (2)
*V* (Å^3^)	2222.2 (5)	4982.9 (12)
*Z*	4	8
Radiation type	Mo *K*α	Mo *K*α
μ (mm^−1^)	0.08	0.08
Crystal size (mm)	0.63 × 0.38 × 0.29	0.50 × 0.19 × 0.13

Data collection		
Diffractometer	Bruker SMART APEXII DUO CCD area-detector	Bruker SMART APEXII DUO CCD area-detector
Absorption correction	Multi-scan (*SADABS*; Bruker, 2009 ▸)	Multi-scan (*SADABS*; Bruker, 2009 ▸)
No. of measured, independent and observed [*I* > 2σ(*I*)] reflections	44210, 5653, 3479	80460, 12643, 5265
*R*_int_	0.047	0.108
(sin θ/λ)_max_ (Å^−1^)	0.673	0.672

Refinement		
*R*[*F* ^2^ > 2σ(*F* ^2^)], *wR*(*F* ^2^), *S*	0.056, 0.142, 1.03	0.084, 0.169, 1.02
No. of reflections	5653	12643
No. of parameters	298	667
H-atom treatment	H-atom parameters constrained	H-atom parameters constrained
Δρ_max_, Δρ_min_ (e Å^−3^)	0.20, −0.18	0.15, −0.15

Computer programs: *APEX2* and *SAINT* (Bruker, 2009 ▸), *SHELXL2014* (Sheldrick, 2015 ▸), *SHELXTL* (Sheldrick, 2008 ▸) and *PLATON* (Spek, 2009 ▸).

## supplementary crystallographic information

### (E)-1-(Anthracen-9-yl)-3-(9-ethyl-9H-carbazol-3-yl)prop-2-en-1-one (mo_DA20_0m) Crystal data

**Table:**

C_31_H_23_NO	*F*(000) = 896
*M**_r_* = 425.50	*D*_x_ = 1.272 Mg m^−^^3^
Monoclinic, *P*2_1_/*c*	Mo *K*α radiation, λ = 0.71073 Å
*a* = 9.3038 (11) Å	Cell parameters from 5727 reflections
*b* = 15.0166 (18) Å	θ = 2.2–22.9°
*c* = 16.1170 (19) Å	µ = 0.08 mm^−^^1^
β = 99.286 (2)°	*T* = 296 K
*V* = 2222.2 (5) Å^3^	Block, yellow
*Z* = 4	0.63 × 0.38 × 0.29 mm

### (E)-1-(Anthracen-9-yl)-3-(9-ethyl-9H-carbazol-3-yl)prop-2-en-1-one (mo_DA20_0m) Data collection

**Table:**

Bruker SMART APEXII DUO CCD area-detector diffractometer	3479 reflections with *I* > 2σ(*I*)
Radiation source: fine-focus sealed tube	*R*_int_ = 0.047
φ and ω scans	θ_max_ = 28.6°, θ_min_ = 1.9°
Absorption correction: multi-scan (*SADABS*; Bruker, 2009)	*h* = −12→11
	*k* = −20→20
44210 measured reflections	*l* = −21→21
5653 independent reflections	

### (E)-1-(Anthracen-9-yl)-3-(9-ethyl-9H-carbazol-3-yl)prop-2-en-1-one (mo_DA20_0m) Refinement

**Table:**

Refinement on *F*^2^	0 restraints
Least-squares matrix: full	Hydrogen site location: inferred from neighbouring sites
*R*[*F*^2^ > 2σ(*F*^2^)] = 0.056	H-atom parameters constrained
*wR*(*F*^2^) = 0.142	*w* = 1/[σ^2^(*F*_o_^2^) + (0.047*P*)^2^ + 0.6503*P*] where *P* = (*F*_o_^2^ + 2*F*_c_^2^)/3
*S* = 1.03	(Δ/σ)_max_ < 0.001
5653 reflections	Δρ_max_ = 0.20 e Å^−^^3^
298 parameters	Δρ_min_ = −0.18 e Å^−^^3^

### (E)-1-(Anthracen-9-yl)-3-(9-ethyl-9H-carbazol-3-yl)prop-2-en-1-one (mo_DA20_0m) Special details

**Table:**

Geometry. All esds (except the esd in the dihedral angle between two l.s. planes) are estimated using the full covariance matrix. The cell esds are taken into account individually in the estimation of esds in distances, angles and torsion angles; correlations between esds in cell parameters are only used when they are defined by crystal symmetry. An approximate (isotropic) treatment of cell esds is used for estimating esds involving l.s. planes.

### (E)-1-(Anthracen-9-yl)-3-(9-ethyl-9H-carbazol-3-yl)prop-2-en-1-one (mo_DA20_0m) Fractional atomic coordinates and isotropic or equivalent isotropic displacement parameters (Å^2^)

**Table:**

	*x*	*y*	*z*	*U*_iso_*/*U*_eq_	
N1	0.30258 (17)	0.43671 (10)	0.60520 (10)	0.0541 (4)	
O1	1.00894 (17)	0.82396 (12)	0.67889 (10)	0.0940 (6)	
C1	0.92274 (19)	0.86663 (11)	0.48174 (11)	0.0484 (4)	
C2	1.0196 (2)	0.79632 (13)	0.46961 (14)	0.0628 (5)	
H2A	1.0445	0.7544	0.5119	0.075*	
C3	1.0762 (2)	0.78928 (16)	0.39769 (16)	0.0734 (6)	
H3A	1.1383	0.7423	0.3909	0.088*	
C4	1.0418 (3)	0.85254 (18)	0.33286 (14)	0.0760 (7)	
H4A	1.0812	0.8469	0.2836	0.091*	
C5	0.9521 (2)	0.92119 (15)	0.34183 (12)	0.0661 (6)	
H5A	0.9315	0.9629	0.2989	0.079*	
C6	0.8883 (2)	0.93085 (12)	0.41595 (11)	0.0509 (4)	
C7	0.7968 (2)	1.00127 (12)	0.42694 (11)	0.0527 (5)	
H7A	0.7763	1.0435	0.3845	0.063*	
C8	0.73470 (19)	1.01073 (11)	0.49924 (11)	0.0476 (4)	
C9	0.6398 (2)	1.08243 (12)	0.51020 (13)	0.0578 (5)	
H9A	0.6192	1.1250	0.4680	0.069*	
C10	0.5788 (2)	1.09027 (14)	0.58016 (14)	0.0659 (5)	
H10A	0.5173	1.1379	0.5859	0.079*	
C11	0.6082 (2)	1.02632 (15)	0.64463 (13)	0.0664 (5)	
H11A	0.5654	1.0320	0.6927	0.080*	
C12	0.6979 (2)	0.95678 (14)	0.63762 (12)	0.0578 (5)	
H12A	0.7153	0.9151	0.6808	0.069*	
C13	0.76632 (18)	0.94624 (11)	0.56498 (10)	0.0464 (4)	
C14	0.86110 (18)	0.87608 (11)	0.55531 (10)	0.0474 (4)	
C15	0.9024 (2)	0.81029 (13)	0.62560 (12)	0.0572 (5)	
C16	0.8125 (2)	0.73143 (12)	0.62919 (12)	0.0567 (5)	
H16A	0.8441	0.6888	0.6699	0.068*	
C17	0.6880 (2)	0.71662 (11)	0.57782 (11)	0.0511 (4)	
H17A	0.6618	0.7581	0.5352	0.061*	
C18	0.5880 (2)	0.64229 (11)	0.58115 (11)	0.0484 (4)	
C19	0.6285 (2)	0.56538 (11)	0.62652 (11)	0.0494 (4)	
H19A	0.7240	0.5585	0.6536	0.059*	
C20	0.52804 (19)	0.49902 (11)	0.63166 (10)	0.0458 (4)	
C21	0.5346 (2)	0.41144 (11)	0.67115 (11)	0.0496 (4)	
C22	0.6445 (2)	0.36261 (13)	0.71850 (12)	0.0609 (5)	
H22A	0.7386	0.3852	0.7306	0.073*	
C23	0.6120 (3)	0.28028 (14)	0.74725 (14)	0.0720 (6)	
H23A	0.6849	0.2471	0.7796	0.086*	
C24	0.4721 (3)	0.24555 (14)	0.72885 (14)	0.0764 (7)	
H24A	0.4536	0.1894	0.7491	0.092*	
C25	0.3595 (3)	0.29223 (13)	0.68122 (13)	0.0644 (5)	
H25A	0.2661	0.2686	0.6686	0.077*	
C26	0.3929 (2)	0.37631 (11)	0.65312 (11)	0.0504 (4)	
C27	0.38240 (19)	0.51092 (11)	0.59169 (11)	0.0481 (4)	
C28	0.3391 (2)	0.58737 (12)	0.54582 (12)	0.0565 (5)	
H28A	0.2431	0.5951	0.5199	0.068*	
C29	0.4432 (2)	0.65129 (12)	0.54014 (11)	0.0555 (5)	
H29A	0.4170	0.7020	0.5082	0.067*	
C30	0.1472 (2)	0.42573 (14)	0.57684 (13)	0.0654 (5)	
H30A	0.1256	0.3628	0.5693	0.079*	
H30B	0.1209	0.4546	0.5227	0.079*	
C31	0.0561 (3)	0.4638 (2)	0.63703 (16)	0.0956 (8)	
H31A	−0.0451	0.4547	0.6153	0.143*	
H31B	0.0799	0.4345	0.6905	0.143*	
H31C	0.0752	0.5264	0.6439	0.143*	

### (E)-1-(Anthracen-9-yl)-3-(9-ethyl-9H-carbazol-3-yl)prop-2-en-1-one (mo_DA20_0m) Atomic displacement parameters (Å^2^)

**Table:**

	*U*^11^	*U*^22^	*U*^33^	*U*^12^	*U*^13^	*U*^23^
N1	0.0529 (9)	0.0477 (8)	0.0619 (9)	−0.0090 (7)	0.0099 (7)	0.0009 (7)
O1	0.0803 (11)	0.0948 (12)	0.0924 (11)	−0.0320 (9)	−0.0302 (9)	0.0327 (9)
C1	0.0446 (10)	0.0435 (9)	0.0558 (10)	−0.0163 (8)	0.0040 (8)	−0.0043 (8)
C2	0.0587 (12)	0.0521 (11)	0.0777 (14)	−0.0129 (9)	0.0110 (10)	−0.0071 (10)
C3	0.0608 (13)	0.0662 (14)	0.0950 (17)	−0.0117 (11)	0.0176 (12)	−0.0257 (13)
C4	0.0717 (15)	0.0934 (18)	0.0668 (13)	−0.0241 (13)	0.0226 (11)	−0.0256 (13)
C5	0.0686 (14)	0.0778 (15)	0.0533 (11)	−0.0207 (12)	0.0139 (10)	−0.0083 (10)
C6	0.0512 (11)	0.0537 (11)	0.0473 (9)	−0.0191 (9)	0.0062 (8)	−0.0035 (8)
C7	0.0568 (11)	0.0516 (11)	0.0477 (10)	−0.0154 (9)	0.0024 (8)	0.0071 (8)
C8	0.0474 (10)	0.0439 (9)	0.0498 (9)	−0.0146 (8)	0.0028 (8)	0.0021 (7)
C9	0.0583 (12)	0.0476 (10)	0.0651 (12)	−0.0085 (9)	0.0028 (9)	0.0028 (9)
C10	0.0599 (13)	0.0615 (13)	0.0763 (14)	−0.0026 (10)	0.0110 (11)	−0.0084 (11)
C11	0.0655 (13)	0.0744 (14)	0.0618 (12)	−0.0089 (11)	0.0179 (10)	−0.0092 (11)
C12	0.0590 (12)	0.0634 (12)	0.0512 (10)	−0.0144 (10)	0.0095 (9)	0.0046 (9)
C13	0.0432 (10)	0.0470 (10)	0.0476 (9)	−0.0157 (8)	0.0034 (7)	0.0011 (8)
C14	0.0454 (10)	0.0460 (9)	0.0488 (9)	−0.0169 (8)	0.0010 (7)	0.0036 (7)
C15	0.0531 (11)	0.0562 (11)	0.0595 (11)	−0.0106 (9)	0.0006 (9)	0.0092 (9)
C16	0.0616 (12)	0.0474 (10)	0.0588 (11)	−0.0067 (9)	0.0027 (9)	0.0122 (8)
C17	0.0599 (11)	0.0419 (9)	0.0523 (10)	−0.0042 (8)	0.0121 (8)	0.0030 (8)
C18	0.0539 (11)	0.0438 (9)	0.0484 (9)	−0.0072 (8)	0.0108 (8)	−0.0023 (8)
C19	0.0507 (10)	0.0461 (10)	0.0519 (10)	−0.0002 (8)	0.0102 (8)	−0.0037 (8)
C20	0.0524 (10)	0.0406 (9)	0.0461 (9)	−0.0039 (8)	0.0134 (8)	−0.0059 (7)
C21	0.0612 (12)	0.0428 (9)	0.0474 (9)	0.0001 (8)	0.0164 (8)	−0.0041 (8)
C22	0.0721 (13)	0.0532 (11)	0.0591 (11)	0.0054 (10)	0.0154 (10)	0.0005 (9)
C23	0.0931 (17)	0.0574 (13)	0.0679 (13)	0.0149 (12)	0.0200 (12)	0.0102 (10)
C24	0.121 (2)	0.0447 (11)	0.0717 (14)	0.0014 (13)	0.0411 (14)	0.0078 (10)
C25	0.0860 (15)	0.0480 (11)	0.0656 (12)	−0.0126 (11)	0.0313 (11)	−0.0029 (10)
C26	0.0640 (12)	0.0420 (9)	0.0486 (9)	−0.0047 (9)	0.0188 (8)	−0.0045 (8)
C27	0.0526 (11)	0.0425 (9)	0.0494 (9)	−0.0056 (8)	0.0090 (8)	−0.0042 (8)
C28	0.0537 (11)	0.0521 (11)	0.0602 (11)	−0.0056 (9)	−0.0015 (9)	−0.0002 (9)
C29	0.0676 (12)	0.0415 (9)	0.0551 (10)	−0.0030 (9)	0.0026 (9)	0.0036 (8)
C30	0.0596 (13)	0.0633 (13)	0.0716 (13)	−0.0168 (10)	0.0052 (10)	0.0008 (10)
C31	0.0596 (15)	0.146 (3)	0.0823 (16)	0.0084 (15)	0.0128 (12)	−0.0006 (17)

### (E)-1-(Anthracen-9-yl)-3-(9-ethyl-9H-carbazol-3-yl)prop-2-en-1-one (mo_DA20_0m) Geometric parameters (Å, º)

**Table:**

N1—C27	1.376 (2)	C16—C17	1.329 (2)
N1—C26	1.384 (2)	C16—H16A	0.9300
N1—C30	1.454 (2)	C17—C18	1.459 (2)
O1—C15	1.220 (2)	C17—H17A	0.9300
C1—C14	1.405 (2)	C18—C19	1.386 (2)
C1—C2	1.422 (3)	C18—C29	1.409 (3)
C1—C6	1.431 (2)	C19—C20	1.378 (2)
C2—C3	1.352 (3)	C19—H19A	0.9300
C2—H2A	0.9300	C20—C27	1.415 (2)
C3—C4	1.410 (3)	C20—C21	1.458 (2)
C3—H3A	0.9300	C21—C22	1.383 (3)
C4—C5	1.349 (3)	C21—C26	1.406 (3)
C4—H4A	0.9300	C22—C23	1.371 (3)
C5—C6	1.424 (3)	C22—H22A	0.9300
C5—H5A	0.9300	C23—C24	1.389 (3)
C6—C7	1.387 (3)	C23—H23A	0.9300
C7—C8	1.389 (2)	C24—C25	1.385 (3)
C7—H7A	0.9300	C24—H24A	0.9300
C8—C9	1.422 (3)	C25—C26	1.393 (2)
C8—C13	1.431 (2)	C25—H25A	0.9300
C9—C10	1.347 (3)	C27—C28	1.390 (2)
C9—H9A	0.9300	C28—C29	1.377 (3)
C10—C11	1.409 (3)	C28—H28A	0.9300
C10—H10A	0.9300	C29—H29A	0.9300
C11—C12	1.353 (3)	C30—C31	1.500 (3)
C11—H11A	0.9300	C30—H30A	0.9700
C12—C13	1.429 (2)	C30—H30B	0.9700
C12—H12A	0.9300	C31—H31A	0.9600
C13—C14	1.399 (2)	C31—H31B	0.9600
C14—C15	1.505 (2)	C31—H31C	0.9600
C15—C16	1.457 (3)		
			
C27—N1—C26	108.92 (15)	C16—C17—C18	126.96 (17)
C27—N1—C30	125.24 (16)	C16—C17—H17A	116.5
C26—N1—C30	125.78 (15)	C18—C17—H17A	116.5
C14—C1—C2	123.03 (17)	C19—C18—C29	119.05 (16)
C14—C1—C6	118.96 (17)	C19—C18—C17	122.42 (17)
C2—C1—C6	118.02 (17)	C29—C18—C17	118.45 (16)
C3—C2—C1	121.3 (2)	C20—C19—C18	120.33 (17)
C3—C2—H2A	119.4	C20—C19—H19A	119.8
C1—C2—H2A	119.4	C18—C19—H19A	119.8
C2—C3—C4	120.6 (2)	C19—C20—C27	119.38 (16)
C2—C3—H3A	119.7	C19—C20—C21	134.24 (17)
C4—C3—H3A	119.7	C27—C20—C21	106.38 (15)
C5—C4—C3	120.4 (2)	C22—C21—C26	120.01 (17)
C5—C4—H4A	119.8	C22—C21—C20	133.93 (18)
C3—C4—H4A	119.8	C26—C21—C20	106.06 (16)
C4—C5—C6	121.1 (2)	C23—C22—C21	118.7 (2)
C4—C5—H5A	119.5	C23—C22—H22A	120.6
C6—C5—H5A	119.5	C21—C22—H22A	120.6
C7—C6—C5	122.16 (18)	C22—C23—C24	121.1 (2)
C7—C6—C1	119.20 (16)	C22—C23—H23A	119.5
C5—C6—C1	118.62 (19)	C24—C23—H23A	119.5
C6—C7—C8	122.16 (17)	C25—C24—C23	121.8 (2)
C6—C7—H7A	118.9	C25—C24—H24A	119.1
C8—C7—H7A	118.9	C23—C24—H24A	119.1
C7—C8—C9	122.18 (17)	C24—C25—C26	116.7 (2)
C7—C8—C13	119.25 (17)	C24—C25—H25A	121.6
C9—C8—C13	118.58 (17)	C26—C25—H25A	121.6
C10—C9—C8	121.62 (19)	N1—C26—C25	128.88 (19)
C10—C9—H9A	119.2	N1—C26—C21	109.52 (15)
C8—C9—H9A	119.2	C25—C26—C21	121.60 (19)
C9—C10—C11	120.0 (2)	N1—C27—C28	129.47 (17)
C9—C10—H10A	120.0	N1—C27—C20	109.11 (15)
C11—C10—H10A	120.0	C28—C27—C20	121.40 (16)
C12—C11—C10	120.90 (19)	C29—C28—C27	117.68 (17)
C12—C11—H11A	119.6	C29—C28—H28A	121.2
C10—C11—H11A	119.5	C27—C28—H28A	121.2
C11—C12—C13	121.09 (18)	C28—C29—C18	122.13 (17)
C11—C12—H12A	119.5	C28—C29—H29A	118.9
C13—C12—H12A	119.5	C18—C29—H29A	118.9
C14—C13—C12	123.17 (16)	N1—C30—C31	112.98 (17)
C14—C13—C8	119.04 (16)	N1—C30—H30A	109.0
C12—C13—C8	117.79 (17)	C31—C30—H30A	109.0
C13—C14—C1	121.38 (16)	N1—C30—H30B	109.0
C13—C14—C15	119.96 (16)	C31—C30—H30B	109.0
C1—C14—C15	118.64 (17)	H30A—C30—H30B	107.8
O1—C15—C16	121.04 (18)	C30—C31—H31A	109.5
O1—C15—C14	119.96 (17)	C30—C31—H31B	109.5
C16—C15—C14	119.00 (16)	H31A—C31—H31B	109.5
C17—C16—C15	124.03 (17)	C30—C31—H31C	109.5
C17—C16—H16A	118.0	H31A—C31—H31C	109.5
C15—C16—H16A	118.0	H31B—C31—H31C	109.5
			
C14—C1—C2—C3	179.71 (17)	C16—C17—C18—C19	−16.4 (3)
C6—C1—C2—C3	−0.9 (3)	C16—C17—C18—C29	160.28 (19)
C1—C2—C3—C4	0.8 (3)	C29—C18—C19—C20	−0.3 (3)
C2—C3—C4—C5	0.2 (3)	C17—C18—C19—C20	176.35 (16)
C3—C4—C5—C6	−1.0 (3)	C18—C19—C20—C27	−1.3 (2)
C4—C5—C6—C7	179.43 (18)	C18—C19—C20—C21	178.28 (17)
C4—C5—C6—C1	0.8 (3)	C19—C20—C21—C22	0.3 (3)
C14—C1—C6—C7	0.9 (2)	C27—C20—C21—C22	179.94 (19)
C2—C1—C6—C7	−178.54 (16)	C19—C20—C21—C26	179.98 (18)
C14—C1—C6—C5	179.53 (15)	C27—C20—C21—C26	−0.37 (18)
C2—C1—C6—C5	0.1 (2)	C26—C21—C22—C23	−0.2 (3)
C5—C6—C7—C8	−179.93 (16)	C20—C21—C22—C23	179.44 (18)
C1—C6—C7—C8	−1.3 (3)	C21—C22—C23—C24	0.6 (3)
C6—C7—C8—C9	−179.38 (16)	C22—C23—C24—C25	−0.2 (3)
C6—C7—C8—C13	0.3 (2)	C23—C24—C25—C26	−0.5 (3)
C7—C8—C9—C10	179.15 (18)	C27—N1—C26—C25	−179.43 (17)
C13—C8—C9—C10	−0.6 (3)	C30—N1—C26—C25	3.2 (3)
C8—C9—C10—C11	−0.2 (3)	C27—N1—C26—C21	−0.17 (19)
C9—C10—C11—C12	0.3 (3)	C30—N1—C26—C21	−177.55 (16)
C10—C11—C12—C13	0.4 (3)	C24—C25—C26—N1	−179.86 (18)
C11—C12—C13—C14	179.25 (17)	C24—C25—C26—C21	1.0 (3)
C11—C12—C13—C8	−1.2 (3)	C22—C21—C26—N1	−179.92 (16)
C7—C8—C13—C14	1.1 (2)	C20—C21—C26—N1	0.33 (18)
C9—C8—C13—C14	−179.17 (15)	C22—C21—C26—C25	−0.6 (3)
C7—C8—C13—C12	−178.51 (15)	C20—C21—C26—C25	179.66 (16)
C9—C8—C13—C12	1.2 (2)	C26—N1—C27—C28	178.40 (18)
C12—C13—C14—C1	178.05 (16)	C30—N1—C27—C28	−4.2 (3)
C8—C13—C14—C1	−1.5 (2)	C26—N1—C27—C20	−0.08 (19)
C12—C13—C14—C15	−3.8 (2)	C30—N1—C27—C20	177.32 (16)
C8—C13—C14—C15	176.65 (15)	C19—C20—C27—N1	180.00 (15)
C2—C1—C14—C13	179.93 (16)	C21—C20—C27—N1	0.28 (18)
C6—C1—C14—C13	0.6 (2)	C19—C20—C27—C28	1.4 (3)
C2—C1—C14—C15	1.7 (2)	C21—C20—C27—C28	−178.35 (16)
C6—C1—C14—C15	−177.65 (15)	N1—C27—C28—C29	−178.00 (17)
C13—C14—C15—O1	−90.0 (2)	C20—C27—C28—C29	0.3 (3)
C1—C14—C15—O1	88.2 (2)	C27—C28—C29—C18	−2.1 (3)
C13—C14—C15—C16	89.2 (2)	C19—C18—C29—C28	2.1 (3)
C1—C14—C15—C16	−92.6 (2)	C17—C18—C29—C28	−174.70 (17)
O1—C15—C16—C17	173.6 (2)	C27—N1—C30—C31	−85.7 (2)
C14—C15—C16—C17	−5.6 (3)	C26—N1—C30—C31	91.2 (2)
C15—C16—C17—C18	−175.80 (18)		

### (E)-1-(Anthracen-9-yl)-3-[4-(9H-carbazol-9-yl)phenyl]prop-2-en-1-one (mo_DA21e_0m) Crystal data

**Table:**

C_35_H_23_NO	*F*(000) = 1984
*M**_r_* = 473.54	*D*_x_ = 1.262 Mg m^−^^3^
Monoclinic, *P*2_1_/*c*	Mo *K*α radiation, λ = 0.71073 Å
*a* = 18.019 (3) Å	Cell parameters from 3880 reflections
*b* = 29.214 (4) Å	θ = 2.3–20.0°
*c* = 9.5503 (13) Å	µ = 0.08 mm^−^^1^
β = 97.637 (2)°	*T* = 296 K
*V* = 4982.9 (12) Å^3^	Plate, yellow
*Z* = 8	0.50 × 0.19 × 0.13 mm

### (E)-1-(Anthracen-9-yl)-3-[4-(9H-carbazol-9-yl)phenyl]prop-2-en-1-one (mo_DA21e_0m) Data collection

**Table:**

Bruker SMART APEXII DUO CCD area-detector diffractometer	5265 reflections with *I* > 2σ(*I*)
Radiation source: fine-focus sealed tube	*R*_int_ = 0.108
φ and ω scans	θ_max_ = 28.5°, θ_min_ = 1.1°
Absorption correction: multi-scan (*SADABS*; Bruker, 2009)	*h* = −24→24
	*k* = −39→39
80460 measured reflections	*l* = −12→12
12643 independent reflections	

### (E)-1-(Anthracen-9-yl)-3-[4-(9H-carbazol-9-yl)phenyl]prop-2-en-1-one (mo_DA21e_0m) Refinement

**Table:**

Refinement on *F*^2^	0 restraints
Least-squares matrix: full	Hydrogen site location: inferred from neighbouring sites
*R*[*F*^2^ > 2σ(*F*^2^)] = 0.084	H-atom parameters constrained
*wR*(*F*^2^) = 0.169	*w* = 1/[σ^2^(*F*_o_^2^) + (0.0387*P*)^2^ + 2.1214*P*] where *P* = (*F*_o_^2^ + 2*F*_c_^2^)/3
*S* = 1.02	(Δ/σ)_max_ < 0.001
12643 reflections	Δρ_max_ = 0.15 e Å^−^^3^
667 parameters	Δρ_min_ = −0.15 e Å^−^^3^

### (E)-1-(Anthracen-9-yl)-3-[4-(9H-carbazol-9-yl)phenyl]prop-2-en-1-one (mo_DA21e_0m) Special details

**Table:**

Geometry. All esds (except the esd in the dihedral angle between two l.s. planes) are estimated using the full covariance matrix. The cell esds are taken into account individually in the estimation of esds in distances, angles and torsion angles; correlations between esds in cell parameters are only used when they are defined by crystal symmetry. An approximate (isotropic) treatment of cell esds is used for estimating esds involving l.s. planes.

### (E)-1-(Anthracen-9-yl)-3-[4-(9H-carbazol-9-yl)phenyl]prop-2-en-1-one (mo_DA21e_0m) Fractional atomic coordinates and isotropic or equivalent isotropic displacement parameters (Å^2^)

**Table:**

	*x*	*y*	*z*	*U*_iso_*/*U*_eq_	
N1A	0.70771 (12)	0.46409 (8)	0.2628 (2)	0.0562 (6)	
O1A	1.07380 (14)	0.46983 (8)	0.9167 (3)	0.1060 (9)	
C1A	1.11780 (15)	0.36157 (10)	0.9308 (3)	0.0550 (7)	
C2A	1.14020 (18)	0.36671 (12)	0.7943 (4)	0.0746 (9)	
H2AA	1.1228	0.3914	0.7378	0.090*	
C3A	1.1869 (2)	0.33565 (16)	0.7463 (4)	0.0971 (12)	
H3AA	1.2003	0.3390	0.6561	0.117*	
C4A	1.2152 (2)	0.29859 (16)	0.8298 (5)	0.0997 (13)	
H4AA	1.2483	0.2783	0.7958	0.120*	
C5A	1.19497 (19)	0.29218 (12)	0.9589 (5)	0.0857 (11)	
H5AA	1.2139	0.2672	1.0128	0.103*	
C6A	1.14504 (16)	0.32293 (10)	1.0144 (3)	0.0630 (8)	
C7A	1.12271 (18)	0.31728 (11)	1.1474 (3)	0.0716 (9)	
H7AA	1.1408	0.2923	1.2021	0.086*	
C8A	1.07435 (17)	0.34759 (10)	1.2011 (3)	0.0618 (8)	
C9A	1.0514 (2)	0.34237 (12)	1.3384 (3)	0.0852 (11)	
H9AA	1.0695	0.3179	1.3952	0.102*	
C10A	1.0038 (2)	0.37244 (14)	1.3864 (4)	0.0945 (12)	
H10A	0.9897	0.3685	1.4759	0.113*	
C11A	0.9754 (2)	0.40949 (12)	1.3031 (4)	0.0855 (11)	
H11A	0.9417	0.4295	1.3369	0.103*	
C12A	0.99626 (18)	0.41645 (10)	1.1742 (3)	0.0669 (8)	
H12A	0.9772	0.4415	1.1210	0.080*	
C13A	1.04704 (15)	0.38610 (9)	1.1177 (3)	0.0531 (7)	
C14A	1.06970 (14)	0.39254 (9)	0.9843 (3)	0.0490 (7)	
C15A	1.04172 (16)	0.43341 (10)	0.8980 (3)	0.0575 (7)	
C16A	0.97565 (15)	0.42839 (10)	0.7923 (3)	0.0572 (7)	
H16A	0.9504	0.4005	0.7855	0.069*	
C17A	0.95048 (14)	0.46190 (9)	0.7064 (3)	0.0517 (7)	
H17A	0.9775	0.4891	0.7176	0.062*	
C18A	0.88611 (14)	0.46221 (9)	0.5960 (2)	0.0461 (6)	
C19A	0.87537 (15)	0.49921 (9)	0.5044 (3)	0.0582 (8)	
H19A	0.9082	0.5239	0.5170	0.070*	
C20A	0.81701 (16)	0.50011 (10)	0.3950 (3)	0.0629 (8)	
H20A	0.8113	0.5250	0.3337	0.075*	
C21A	0.76707 (14)	0.46405 (9)	0.3766 (3)	0.0486 (7)	
C22A	0.77644 (15)	0.42742 (9)	0.4683 (3)	0.0524 (7)	
H22A	0.7428	0.4031	0.4571	0.063*	
C23A	0.83510 (15)	0.42646 (9)	0.5763 (3)	0.0522 (7)	
H23A	0.8407	0.4015	0.6370	0.063*	
C24A	0.71655 (16)	0.46292 (9)	0.1192 (3)	0.0527 (7)	
C25A	0.78105 (17)	0.46512 (10)	0.0569 (3)	0.0629 (8)	
H25A	0.8278	0.4673	0.1110	0.075*	
C26A	0.7740 (2)	0.46406 (11)	−0.0876 (3)	0.0766 (9)	
H26A	0.8169	0.4656	−0.1320	0.092*	
C27A	0.7054 (2)	0.46071 (11)	−0.1686 (3)	0.0818 (10)	
H27A	0.7028	0.4605	−0.2665	0.098*	
C28A	0.6409 (2)	0.45763 (10)	−0.1087 (3)	0.0736 (9)	
H28A	0.5947	0.4549	−0.1645	0.088*	
C29A	0.64597 (16)	0.45865 (9)	0.0391 (3)	0.0562 (7)	
C30A	0.59190 (16)	0.45608 (9)	0.1376 (3)	0.0596 (8)	
C31A	0.51464 (19)	0.45214 (11)	0.1233 (4)	0.0817 (10)	
H31A	0.4871	0.4494	0.0341	0.098*	
C32A	0.4791 (2)	0.45237 (11)	0.2415 (5)	0.0918 (12)	
H32A	0.4274	0.4490	0.2318	0.110*	
C33A	0.5186 (2)	0.45750 (11)	0.3751 (5)	0.0856 (11)	
H33A	0.4929	0.4581	0.4532	0.103*	
C34A	0.59657 (18)	0.46183 (10)	0.3946 (4)	0.0707 (9)	
H34A	0.6235	0.4658	0.4838	0.085*	
C35A	0.63152 (16)	0.45997 (9)	0.2741 (3)	0.0573 (7)	
N1B	0.75504 (13)	0.31672 (7)	0.7498 (2)	0.0545 (6)	
O1B	0.39724 (14)	0.25060 (8)	0.0493 (3)	0.1037 (9)	
C1B	0.38794 (16)	0.35263 (10)	−0.0405 (3)	0.0588 (8)	
C2B	0.4305 (2)	0.33541 (13)	−0.1434 (4)	0.0877 (11)	
H2BA	0.4566	0.3081	−0.1266	0.105*	
C3B	0.4336 (3)	0.35821 (17)	−0.2660 (4)	0.1139 (14)	
H3BA	0.4606	0.3459	−0.3334	0.137*	
C4B	0.3965 (3)	0.40018 (16)	−0.2928 (4)	0.1061 (13)	
H4BA	0.4003	0.4158	−0.3763	0.127*	
C5B	0.3555 (2)	0.41782 (12)	−0.1987 (4)	0.0840 (10)	
H5BA	0.3311	0.4456	−0.2181	0.101*	
C6B	0.34842 (17)	0.39491 (10)	−0.0695 (3)	0.0614 (8)	
C7B	0.30545 (17)	0.41207 (10)	0.0289 (3)	0.0661 (8)	
H7BA	0.2797	0.4394	0.0095	0.079*	
C8B	0.29961 (16)	0.38992 (10)	0.1545 (3)	0.0600 (8)	
C9B	0.25652 (18)	0.40812 (12)	0.2558 (4)	0.0821 (10)	
H9BA	0.2306	0.4355	0.2373	0.099*	
C10B	0.2529 (2)	0.38588 (15)	0.3791 (4)	0.0962 (12)	
H10B	0.2253	0.3984	0.4452	0.115*	
C11B	0.2903 (2)	0.34412 (14)	0.4084 (4)	0.0887 (11)	
H11B	0.2870	0.3293	0.4935	0.106*	
C12B	0.33094 (17)	0.32540 (11)	0.3147 (3)	0.0686 (8)	
H12B	0.3545	0.2974	0.3352	0.082*	
C13B	0.33845 (15)	0.34761 (9)	0.1845 (3)	0.0533 (7)	
C14B	0.38207 (15)	0.32977 (9)	0.0867 (3)	0.0530 (7)	
C15B	0.42182 (17)	0.28463 (10)	0.1130 (3)	0.0627 (8)	
C16B	0.48985 (16)	0.28167 (10)	0.2149 (3)	0.0619 (8)	
H16B	0.5164	0.2543	0.2208	0.074*	
C17B	0.51593 (15)	0.31550 (10)	0.2990 (3)	0.0565 (7)	
H17B	0.4913	0.3434	0.2839	0.068*	
C18B	0.57872 (15)	0.31449 (9)	0.4128 (3)	0.0525 (7)	
C19B	0.62943 (16)	0.27845 (10)	0.4343 (3)	0.0636 (8)	
H19B	0.6250	0.2537	0.3724	0.076*	
C20B	0.68616 (16)	0.27895 (9)	0.5463 (3)	0.0609 (8)	
H20B	0.7189	0.2543	0.5606	0.073*	
C21B	0.69482 (15)	0.31594 (9)	0.6380 (3)	0.0513 (7)	
C22B	0.64470 (16)	0.35165 (9)	0.6181 (3)	0.0586 (8)	
H22B	0.6495	0.3765	0.6796	0.070*	
C23B	0.58778 (15)	0.35067 (9)	0.5079 (3)	0.0586 (8)	
H23B	0.5542	0.3750	0.4963	0.070*	
C24B	0.82957 (16)	0.30586 (9)	0.7360 (3)	0.0538 (7)	
C25B	0.86218 (18)	0.29651 (10)	0.6159 (3)	0.0651 (8)	
H25B	0.8338	0.2960	0.5271	0.078*	
C26B	0.93814 (19)	0.28801 (10)	0.6323 (4)	0.0746 (9)	
H26B	0.9611	0.2813	0.5530	0.089*	
C27B	0.98123 (19)	0.28918 (10)	0.7642 (4)	0.0788 (10)	
H27B	1.0322	0.2829	0.7722	0.095*	
C28B	0.94911 (19)	0.29957 (10)	0.8828 (4)	0.0728 (9)	
H28B	0.9781	0.3008	0.9708	0.087*	
C29B	0.87234 (17)	0.30820 (9)	0.8695 (3)	0.0584 (8)	
C30B	0.82245 (18)	0.32079 (9)	0.9691 (3)	0.0606 (8)	
C31B	0.8318 (2)	0.32666 (11)	1.1151 (3)	0.0823 (10)	
H31B	0.8786	0.3231	1.1679	0.099*	
C32B	0.7706 (3)	0.33779 (12)	1.1797 (4)	0.0923 (12)	
H32B	0.7765	0.3415	1.2773	0.111*	
C33B	0.7005 (2)	0.34362 (11)	1.1031 (4)	0.0844 (11)	
H33B	0.6603	0.3516	1.1500	0.101*	
C34B	0.68903 (19)	0.33782 (9)	0.9578 (3)	0.0667 (8)	
H34B	0.6419	0.3416	0.9060	0.080*	
C35B	0.75101 (17)	0.32615 (9)	0.8929 (3)	0.0559 (7)	

### (E)-1-(Anthracen-9-yl)-3-[4-(9H-carbazol-9-yl)phenyl]prop-2-en-1-one (mo_DA21e_0m) Atomic displacement parameters (Å^2^)

**Table:**

	*U*^11^	*U*^22^	*U*^33^	*U*^12^	*U*^13^	*U*^23^
N1A	0.0463 (14)	0.0716 (16)	0.0486 (14)	−0.0026 (12)	−0.0021 (11)	0.0073 (12)
O1A	0.1001 (19)	0.0713 (16)	0.130 (2)	−0.0338 (14)	−0.0479 (15)	0.0325 (14)
C1A	0.0367 (16)	0.0623 (19)	0.0628 (19)	−0.0078 (14)	−0.0048 (14)	−0.0074 (15)
C2A	0.058 (2)	0.088 (3)	0.079 (2)	−0.0115 (18)	0.0133 (18)	−0.0093 (19)
C3A	0.073 (3)	0.121 (4)	0.102 (3)	−0.020 (3)	0.031 (2)	−0.029 (3)
C4A	0.054 (2)	0.107 (3)	0.138 (4)	0.000 (2)	0.013 (3)	−0.043 (3)
C5A	0.056 (2)	0.079 (3)	0.116 (3)	0.0077 (19)	−0.015 (2)	−0.016 (2)
C6A	0.0424 (18)	0.060 (2)	0.080 (2)	0.0045 (15)	−0.0160 (16)	−0.0102 (17)
C7A	0.072 (2)	0.060 (2)	0.074 (2)	0.0050 (17)	−0.0213 (18)	0.0111 (17)
C8A	0.068 (2)	0.0567 (19)	0.0560 (19)	−0.0047 (16)	−0.0090 (15)	0.0053 (15)
C9A	0.118 (3)	0.073 (2)	0.061 (2)	−0.010 (2)	−0.002 (2)	0.0162 (18)
C10A	0.129 (4)	0.091 (3)	0.067 (2)	−0.017 (3)	0.025 (2)	−0.005 (2)
C11A	0.108 (3)	0.075 (2)	0.079 (2)	−0.007 (2)	0.031 (2)	−0.012 (2)
C12A	0.076 (2)	0.058 (2)	0.066 (2)	−0.0043 (17)	0.0100 (17)	−0.0042 (16)
C13A	0.0508 (18)	0.0528 (18)	0.0524 (17)	−0.0060 (14)	−0.0055 (14)	−0.0015 (14)
C14A	0.0402 (16)	0.0515 (17)	0.0515 (17)	−0.0037 (13)	−0.0076 (13)	0.0024 (13)
C15A	0.0518 (18)	0.0578 (19)	0.0601 (18)	−0.0069 (15)	−0.0035 (14)	0.0063 (15)
C16A	0.0577 (19)	0.0539 (18)	0.0558 (17)	−0.0051 (14)	−0.0077 (14)	0.0059 (14)
C17A	0.0538 (17)	0.0520 (17)	0.0475 (16)	−0.0033 (14)	0.0003 (13)	0.0027 (13)
C18A	0.0504 (16)	0.0469 (16)	0.0397 (14)	0.0011 (13)	0.0019 (12)	0.0006 (12)
C19A	0.0564 (18)	0.0504 (17)	0.0632 (18)	−0.0088 (14)	−0.0093 (15)	0.0103 (14)
C20A	0.061 (2)	0.0593 (19)	0.0646 (19)	−0.0017 (16)	−0.0059 (15)	0.0182 (15)
C21A	0.0454 (16)	0.0554 (17)	0.0433 (15)	0.0017 (14)	−0.0006 (12)	0.0038 (13)
C22A	0.0547 (18)	0.0533 (18)	0.0478 (16)	−0.0077 (13)	0.0017 (14)	0.0024 (13)
C23A	0.0621 (19)	0.0489 (17)	0.0438 (15)	−0.0030 (14)	0.0004 (14)	0.0059 (12)
C24A	0.0521 (18)	0.0509 (17)	0.0523 (17)	0.0012 (14)	−0.0039 (14)	0.0026 (13)
C25A	0.057 (2)	0.072 (2)	0.0572 (19)	−0.0008 (16)	0.0004 (15)	0.0037 (15)
C26A	0.090 (3)	0.080 (2)	0.060 (2)	0.0094 (19)	0.0113 (19)	−0.0016 (17)
C27A	0.110 (3)	0.077 (2)	0.056 (2)	0.013 (2)	0.002 (2)	−0.0061 (17)
C28A	0.086 (3)	0.058 (2)	0.067 (2)	0.0068 (18)	−0.0259 (19)	−0.0079 (16)
C29A	0.0574 (19)	0.0443 (17)	0.0625 (19)	0.0033 (14)	−0.0083 (15)	0.0012 (14)
C30A	0.0451 (18)	0.0448 (17)	0.083 (2)	0.0002 (13)	−0.0147 (16)	0.0057 (15)
C31A	0.060 (2)	0.060 (2)	0.119 (3)	−0.0025 (17)	−0.013 (2)	0.008 (2)
C32A	0.049 (2)	0.067 (2)	0.157 (4)	−0.0054 (18)	0.002 (3)	0.020 (3)
C33A	0.067 (2)	0.066 (2)	0.129 (3)	0.0064 (19)	0.032 (2)	0.024 (2)
C34A	0.058 (2)	0.073 (2)	0.082 (2)	0.0024 (17)	0.0120 (18)	0.0180 (17)
C35A	0.0462 (18)	0.0521 (18)	0.072 (2)	0.0016 (14)	0.0019 (16)	0.0111 (15)
N1B	0.0536 (16)	0.0558 (14)	0.0519 (14)	−0.0008 (12)	−0.0010 (12)	0.0017 (11)
O1B	0.126 (2)	0.0615 (15)	0.1070 (19)	0.0105 (14)	−0.0459 (16)	−0.0209 (14)
C1B	0.0581 (19)	0.0586 (19)	0.0557 (18)	−0.0037 (15)	−0.0066 (15)	−0.0021 (15)
C2B	0.097 (3)	0.092 (3)	0.076 (2)	0.011 (2)	0.019 (2)	0.004 (2)
C3B	0.137 (4)	0.124 (4)	0.087 (3)	0.010 (3)	0.039 (3)	0.009 (3)
C4B	0.135 (4)	0.111 (4)	0.073 (3)	−0.015 (3)	0.015 (3)	0.022 (2)
C5B	0.096 (3)	0.073 (2)	0.078 (2)	−0.011 (2)	−0.007 (2)	0.017 (2)
C6B	0.061 (2)	0.0554 (19)	0.0617 (19)	−0.0103 (16)	−0.0148 (16)	0.0044 (16)
C7B	0.059 (2)	0.0495 (18)	0.083 (2)	0.0007 (15)	−0.0130 (18)	0.0011 (17)
C8B	0.0497 (18)	0.0560 (19)	0.071 (2)	−0.0037 (15)	−0.0048 (15)	−0.0027 (16)
C9B	0.064 (2)	0.074 (2)	0.108 (3)	0.0090 (18)	0.011 (2)	−0.006 (2)
C10B	0.089 (3)	0.107 (3)	0.098 (3)	0.008 (2)	0.032 (2)	−0.008 (3)
C11B	0.089 (3)	0.104 (3)	0.077 (2)	0.002 (2)	0.022 (2)	0.007 (2)
C12B	0.063 (2)	0.072 (2)	0.068 (2)	−0.0015 (17)	0.0006 (17)	0.0072 (18)
C13B	0.0456 (17)	0.0513 (17)	0.0591 (18)	−0.0038 (14)	−0.0082 (14)	0.0009 (14)
C14B	0.0460 (17)	0.0538 (17)	0.0547 (17)	0.0010 (14)	−0.0105 (14)	−0.0036 (14)
C15B	0.067 (2)	0.0548 (19)	0.0621 (19)	0.0009 (16)	−0.0084 (16)	−0.0054 (15)
C16B	0.062 (2)	0.0519 (18)	0.067 (2)	0.0092 (15)	−0.0061 (16)	−0.0007 (15)
C17B	0.0528 (18)	0.0500 (17)	0.0644 (19)	0.0072 (14)	−0.0001 (14)	0.0056 (14)
C18B	0.0479 (17)	0.0489 (17)	0.0580 (17)	0.0019 (14)	−0.0030 (14)	0.0044 (14)
C19B	0.066 (2)	0.0551 (18)	0.0646 (19)	0.0102 (15)	−0.0100 (16)	−0.0102 (15)
C20B	0.061 (2)	0.0496 (18)	0.0676 (19)	0.0130 (14)	−0.0081 (16)	−0.0032 (15)
C21B	0.0539 (18)	0.0463 (17)	0.0516 (16)	−0.0017 (14)	−0.0002 (14)	0.0035 (13)
C22B	0.062 (2)	0.0451 (17)	0.0659 (19)	0.0014 (15)	−0.0013 (16)	−0.0038 (14)
C23B	0.0524 (18)	0.0447 (17)	0.076 (2)	0.0069 (13)	−0.0035 (15)	0.0026 (15)
C24B	0.0533 (19)	0.0454 (16)	0.0606 (19)	−0.0061 (14)	−0.0002 (15)	0.0042 (14)
C25B	0.066 (2)	0.0599 (19)	0.068 (2)	−0.0054 (16)	0.0038 (17)	0.0018 (15)
C26B	0.068 (2)	0.064 (2)	0.094 (3)	−0.0066 (18)	0.020 (2)	−0.0039 (18)
C27B	0.059 (2)	0.061 (2)	0.113 (3)	−0.0075 (17)	−0.001 (2)	−0.003 (2)
C28B	0.065 (2)	0.058 (2)	0.087 (2)	−0.0115 (17)	−0.0179 (19)	−0.0019 (18)
C29B	0.060 (2)	0.0478 (17)	0.064 (2)	−0.0098 (15)	−0.0036 (16)	−0.0001 (14)
C30B	0.073 (2)	0.0443 (17)	0.0590 (19)	−0.0113 (15)	−0.0111 (17)	−0.0030 (14)
C31B	0.107 (3)	0.071 (2)	0.062 (2)	−0.007 (2)	−0.015 (2)	−0.0104 (18)
C32B	0.146 (4)	0.073 (2)	0.057 (2)	0.006 (2)	0.010 (3)	−0.0099 (18)
C33B	0.124 (3)	0.060 (2)	0.074 (3)	0.011 (2)	0.032 (2)	0.0011 (18)
C34B	0.082 (2)	0.0485 (18)	0.071 (2)	0.0028 (16)	0.0127 (18)	0.0013 (15)
C35B	0.072 (2)	0.0416 (16)	0.0535 (18)	−0.0052 (15)	0.0054 (16)	−0.0008 (13)

### (E)-1-(Anthracen-9-yl)-3-[4-(9H-carbazol-9-yl)phenyl]prop-2-en-1-one (mo_DA21e_0m) Geometric parameters (Å, º)

**Table:**

N1A—C35A	1.397 (3)	N1B—C24B	1.403 (3)
N1A—C24A	1.402 (3)	N1B—C35B	1.405 (3)
N1A—C21A	1.420 (3)	N1B—C21B	1.418 (3)
O1A—C15A	1.213 (3)	O1B—C15B	1.218 (3)
C1A—C14A	1.396 (4)	C1B—C14B	1.402 (4)
C1A—C2A	1.423 (4)	C1B—C2B	1.416 (4)
C1A—C6A	1.431 (4)	C1B—C6B	1.434 (4)
C2A—C3A	1.358 (5)	C2B—C3B	1.355 (5)
C2A—H2AA	0.9300	C2B—H2BA	0.9300
C3A—C4A	1.401 (5)	C3B—C4B	1.404 (5)
C3A—H3AA	0.9300	C3B—H3BA	0.9300
C4A—C5A	1.344 (5)	C4B—C5B	1.340 (5)
C4A—H4AA	0.9300	C4B—H4BA	0.9300
C5A—C6A	1.423 (4)	C5B—C6B	1.424 (4)
C5A—H5AA	0.9300	C5B—H5BA	0.9300
C6A—C7A	1.392 (4)	C6B—C7B	1.388 (4)
C7A—C8A	1.388 (4)	C7B—C8B	1.379 (4)
C7A—H7AA	0.9300	C7B—H7BA	0.9300
C8A—C13A	1.428 (4)	C8B—C9B	1.422 (4)
C8A—C9A	1.435 (4)	C8B—C13B	1.431 (4)
C9A—C10A	1.350 (5)	C9B—C10B	1.354 (5)
C9A—H9AA	0.9300	C9B—H9BA	0.9300
C10A—C11A	1.400 (5)	C10B—C11B	1.404 (5)
C10A—H10A	0.9300	C10B—H10B	0.9300
C11A—C12A	1.350 (4)	C11B—C12B	1.345 (4)
C11A—H11A	0.9300	C11B—H11B	0.9300
C12A—C13A	1.430 (4)	C12B—C13B	1.424 (4)
C12A—H12A	0.9300	C12B—H12B	0.9300
C13A—C14A	1.401 (4)	C13B—C14B	1.399 (4)
C14A—C15A	1.500 (4)	C14B—C15B	1.506 (4)
C15A—C16A	1.462 (4)	C15B—C16B	1.463 (4)
C16A—C17A	1.319 (3)	C16B—C17B	1.320 (4)
C16A—H16A	0.9300	C16B—H16B	0.9300
C17A—C18A	1.460 (3)	C17B—C18B	1.461 (4)
C17A—H17A	0.9300	C17B—H17B	0.9300
C18A—C23A	1.387 (3)	C18B—C23B	1.389 (4)
C18A—C19A	1.388 (3)	C18B—C19B	1.391 (4)
C19A—C20A	1.381 (4)	C19B—C20B	1.378 (4)
C19A—H19A	0.9300	C19B—H19B	0.9300
C20A—C21A	1.381 (4)	C20B—C21B	1.386 (4)
C20A—H20A	0.9300	C20B—H20B	0.9300
C21A—C22A	1.379 (3)	C21B—C22B	1.376 (4)
C22A—C23A	1.375 (3)	C22B—C23B	1.369 (4)
C22A—H22A	0.9300	C22B—H22B	0.9300
C23A—H23A	0.9300	C23B—H23B	0.9300
C24A—C25A	1.376 (4)	C24B—C25B	1.383 (4)
C24A—C29A	1.400 (4)	C24B—C29B	1.401 (4)
C25A—C26A	1.369 (4)	C25B—C26B	1.379 (4)
C25A—H25A	0.9300	C25B—H25B	0.9300
C26A—C27A	1.371 (4)	C26B—C27B	1.390 (4)
C26A—H26A	0.9300	C26B—H26B	0.9300
C27A—C28A	1.366 (4)	C27B—C28B	1.372 (4)
C27A—H27A	0.9300	C27B—H27B	0.9300
C28A—C29A	1.403 (4)	C28B—C29B	1.395 (4)
C28A—H28A	0.9300	C28B—H28B	0.9300
C29A—C30A	1.443 (4)	C29B—C30B	1.441 (4)
C30A—C31A	1.386 (4)	C30B—C31B	1.392 (4)
C30A—C35A	1.405 (4)	C30B—C35B	1.401 (4)
C31A—C32A	1.370 (5)	C31B—C32B	1.371 (5)
C31A—H31A	0.9300	C31B—H31B	0.9300
C32A—C33A	1.384 (5)	C32B—C33B	1.384 (5)
C32A—H32A	0.9300	C32B—H32B	0.9300
C33A—C34A	1.398 (4)	C33B—C34B	1.386 (4)
C33A—H33A	0.9300	C33B—H33B	0.9300
C34A—C35A	1.385 (4)	C34B—C35B	1.390 (4)
C34A—H34A	0.9300	C34B—H34B	0.9300
			
C35A—N1A—C24A	108.3 (2)	C24B—N1B—C35B	108.0 (2)
C35A—N1A—C21A	126.1 (2)	C24B—N1B—C21B	124.9 (2)
C24A—N1A—C21A	125.2 (2)	C35B—N1B—C21B	127.0 (2)
C14A—C1A—C2A	122.2 (3)	C14B—C1B—C2B	122.8 (3)
C14A—C1A—C6A	119.4 (3)	C14B—C1B—C6B	119.0 (3)
C2A—C1A—C6A	118.5 (3)	C2B—C1B—C6B	118.2 (3)
C3A—C2A—C1A	120.2 (4)	C3B—C2B—C1B	120.9 (4)
C3A—C2A—H2AA	119.9	C3B—C2B—H2BA	119.6
C1A—C2A—H2AA	119.9	C1B—C2B—H2BA	119.6
C2A—C3A—C4A	121.3 (4)	C2B—C3B—C4B	120.9 (4)
C2A—C3A—H3AA	119.3	C2B—C3B—H3BA	119.5
C4A—C3A—H3AA	119.3	C4B—C3B—H3BA	119.5
C5A—C4A—C3A	120.5 (4)	C5B—C4B—C3B	120.3 (4)
C5A—C4A—H4AA	119.8	C5B—C4B—H4BA	119.8
C3A—C4A—H4AA	119.8	C3B—C4B—H4BA	119.8
C4A—C5A—C6A	121.1 (4)	C4B—C5B—C6B	121.4 (4)
C4A—C5A—H5AA	119.5	C4B—C5B—H5BA	119.3
C6A—C5A—H5AA	119.5	C6B—C5B—H5BA	119.3
C7A—C6A—C5A	122.6 (3)	C7B—C6B—C5B	122.6 (3)
C7A—C6A—C1A	118.9 (3)	C7B—C6B—C1B	119.2 (3)
C5A—C6A—C1A	118.5 (3)	C5B—C6B—C1B	118.2 (3)
C8A—C7A—C6A	122.2 (3)	C8B—C7B—C6B	122.2 (3)
C8A—C7A—H7AA	118.9	C8B—C7B—H7BA	118.9
C6A—C7A—H7AA	118.9	C6B—C7B—H7BA	118.9
C7A—C8A—C13A	118.9 (3)	C7B—C8B—C9B	121.9 (3)
C7A—C8A—C9A	122.8 (3)	C7B—C8B—C13B	119.2 (3)
C13A—C8A—C9A	118.3 (3)	C9B—C8B—C13B	118.9 (3)
C10A—C9A—C8A	121.0 (3)	C10B—C9B—C8B	120.4 (3)
C10A—C9A—H9AA	119.5	C10B—C9B—H9BA	119.8
C8A—C9A—H9AA	119.5	C8B—C9B—H9BA	119.8
C9A—C10A—C11A	120.7 (3)	C9B—C10B—C11B	120.9 (4)
C9A—C10A—H10A	119.6	C9B—C10B—H10B	119.5
C11A—C10A—H10A	119.6	C11B—C10B—H10B	119.5
C12A—C11A—C10A	120.6 (3)	C12B—C11B—C10B	120.6 (3)
C12A—C11A—H11A	119.7	C12B—C11B—H11B	119.7
C10A—C11A—H11A	119.7	C10B—C11B—H11B	119.7
C11A—C12A—C13A	121.4 (3)	C11B—C12B—C13B	121.2 (3)
C11A—C12A—H12A	119.3	C11B—C12B—H12B	119.4
C13A—C12A—H12A	119.3	C13B—C12B—H12B	119.4
C14A—C13A—C8A	119.5 (3)	C14B—C13B—C12B	122.7 (3)
C14A—C13A—C12A	122.6 (3)	C14B—C13B—C8B	119.4 (3)
C8A—C13A—C12A	117.9 (3)	C12B—C13B—C8B	117.9 (3)
C1A—C14A—C13A	121.1 (2)	C13B—C14B—C1B	121.0 (3)
C1A—C14A—C15A	119.7 (3)	C13B—C14B—C15B	120.8 (3)
C13A—C14A—C15A	119.3 (3)	C1B—C14B—C15B	118.2 (3)
O1A—C15A—C16A	121.2 (3)	O1B—C15B—C16B	120.1 (3)
O1A—C15A—C14A	119.9 (2)	O1B—C15B—C14B	119.9 (3)
C16A—C15A—C14A	118.9 (2)	C16B—C15B—C14B	120.0 (3)
C17A—C16A—C15A	122.4 (3)	C17B—C16B—C15B	124.0 (3)
C17A—C16A—H16A	118.8	C17B—C16B—H16B	118.0
C15A—C16A—H16A	118.8	C15B—C16B—H16B	118.0
C16A—C17A—C18A	129.0 (3)	C16B—C17B—C18B	128.0 (3)
C16A—C17A—H17A	115.5	C16B—C17B—H17B	116.0
C18A—C17A—H17A	115.5	C18B—C17B—H17B	116.0
C23A—C18A—C19A	117.8 (2)	C23B—C18B—C19B	117.5 (2)
C23A—C18A—C17A	122.8 (2)	C23B—C18B—C17B	118.9 (2)
C19A—C18A—C17A	119.4 (2)	C19B—C18B—C17B	123.6 (3)
C20A—C19A—C18A	121.3 (3)	C20B—C19B—C18B	120.8 (3)
C20A—C19A—H19A	119.3	C20B—C19B—H19B	119.6
C18A—C19A—H19A	119.3	C18B—C19B—H19B	119.6
C19A—C20A—C21A	120.0 (3)	C19B—C20B—C21B	120.5 (3)
C19A—C20A—H20A	120.0	C19B—C20B—H20B	119.7
C21A—C20A—H20A	120.0	C21B—C20B—H20B	119.7
C22A—C21A—C20A	119.2 (2)	C22B—C21B—C20B	119.1 (2)
C22A—C21A—N1A	120.2 (2)	C22B—C21B—N1B	120.9 (2)
C20A—C21A—N1A	120.6 (2)	C20B—C21B—N1B	119.9 (2)
C23A—C22A—C21A	120.6 (3)	C23B—C22B—C21B	120.1 (3)
C23A—C22A—H22A	119.7	C23B—C22B—H22B	120.0
C21A—C22A—H22A	119.7	C21B—C22B—H22B	120.0
C22A—C23A—C18A	121.0 (2)	C22B—C23B—C18B	122.0 (3)
C22A—C23A—H23A	119.5	C22B—C23B—H23B	119.0
C18A—C23A—H23A	119.5	C18B—C23B—H23B	119.0
C25A—C24A—C29A	121.8 (3)	C25B—C24B—C29B	121.4 (3)
C25A—C24A—N1A	129.4 (2)	C25B—C24B—N1B	129.8 (3)
C29A—C24A—N1A	108.9 (3)	C29B—C24B—N1B	108.7 (3)
C26A—C25A—C24A	117.7 (3)	C26B—C25B—C24B	117.8 (3)
C26A—C25A—H25A	121.2	C26B—C25B—H25B	121.1
C24A—C25A—H25A	121.2	C24B—C25B—H25B	121.1
C25A—C26A—C27A	121.7 (3)	C25B—C26B—C27B	121.6 (3)
C25A—C26A—H26A	119.1	C25B—C26B—H26B	119.2
C27A—C26A—H26A	119.1	C27B—C26B—H26B	119.2
C28A—C27A—C26A	121.5 (3)	C28B—C27B—C26B	120.5 (3)
C28A—C27A—H27A	119.3	C28B—C27B—H27B	119.8
C26A—C27A—H27A	119.3	C26B—C27B—H27B	119.8
C27A—C28A—C29A	118.3 (3)	C27B—C28B—C29B	119.1 (3)
C27A—C28A—H28A	120.8	C27B—C28B—H28B	120.4
C29A—C28A—H28A	120.8	C29B—C28B—H28B	120.4
C24A—C29A—C28A	119.0 (3)	C28B—C29B—C24B	119.5 (3)
C24A—C29A—C30A	107.0 (2)	C28B—C29B—C30B	133.2 (3)
C28A—C29A—C30A	134.0 (3)	C24B—C29B—C30B	107.3 (3)
C31A—C30A—C35A	118.6 (3)	C31B—C30B—C35B	119.1 (3)
C31A—C30A—C29A	134.2 (3)	C31B—C30B—C29B	133.6 (3)
C35A—C30A—C29A	107.3 (2)	C35B—C30B—C29B	107.2 (3)
C32A—C31A—C30A	119.4 (3)	C32B—C31B—C30B	118.9 (3)
C32A—C31A—H31A	120.3	C32B—C31B—H31B	120.6
C30A—C31A—H31A	120.3	C30B—C31B—H31B	120.6
C31A—C32A—C33A	121.4 (3)	C31B—C32B—C33B	121.5 (3)
C31A—C32A—H32A	119.3	C31B—C32B—H32B	119.2
C33A—C32A—H32A	119.3	C33B—C32B—H32B	119.2
C32A—C33A—C34A	121.1 (3)	C32B—C33B—C34B	121.2 (3)
C32A—C33A—H33A	119.4	C32B—C33B—H33B	119.4
C34A—C33A—H33A	119.4	C34B—C33B—H33B	119.4
C35A—C34A—C33A	116.5 (3)	C33B—C34B—C35B	117.1 (3)
C35A—C34A—H34A	121.8	C33B—C34B—H34B	121.5
C33A—C34A—H34A	121.8	C35B—C34B—H34B	121.5
C34A—C35A—N1A	128.4 (3)	C34B—C35B—C30B	122.2 (3)
C34A—C35A—C30A	122.9 (3)	C34B—C35B—N1B	129.1 (3)
N1A—C35A—C30A	108.6 (3)	C30B—C35B—N1B	108.7 (3)
			
C14A—C1A—C2A—C3A	−179.8 (3)	C14B—C1B—C2B—C3B	178.8 (3)
C6A—C1A—C2A—C3A	−0.6 (4)	C6B—C1B—C2B—C3B	−0.2 (5)
C1A—C2A—C3A—C4A	−1.3 (5)	C1B—C2B—C3B—C4B	1.8 (6)
C2A—C3A—C4A—C5A	1.9 (6)	C2B—C3B—C4B—C5B	−1.9 (7)
C3A—C4A—C5A—C6A	−0.6 (5)	C3B—C4B—C5B—C6B	0.2 (6)
C4A—C5A—C6A—C7A	179.8 (3)	C4B—C5B—C6B—C7B	−179.0 (3)
C4A—C5A—C6A—C1A	−1.2 (5)	C4B—C5B—C6B—C1B	1.4 (5)
C14A—C1A—C6A—C7A	0.0 (4)	C14B—C1B—C6B—C7B	0.0 (4)
C2A—C1A—C6A—C7A	−179.2 (3)	C2B—C1B—C6B—C7B	179.0 (3)
C14A—C1A—C6A—C5A	−179.0 (3)	C14B—C1B—C6B—C5B	179.5 (3)
C2A—C1A—C6A—C5A	1.8 (4)	C2B—C1B—C6B—C5B	−1.4 (4)
C5A—C6A—C7A—C8A	179.6 (3)	C5B—C6B—C7B—C8B	−179.2 (3)
C1A—C6A—C7A—C8A	0.6 (4)	C1B—C6B—C7B—C8B	0.3 (4)
C6A—C7A—C8A—C13A	−0.5 (4)	C6B—C7B—C8B—C9B	178.9 (3)
C6A—C7A—C8A—C9A	−179.5 (3)	C6B—C7B—C8B—C13B	−0.6 (4)
C7A—C8A—C9A—C10A	−179.5 (3)	C7B—C8B—C9B—C10B	−178.9 (3)
C13A—C8A—C9A—C10A	1.5 (5)	C13B—C8B—C9B—C10B	0.6 (5)
C8A—C9A—C10A—C11A	0.2 (6)	C8B—C9B—C10B—C11B	−1.2 (6)
C9A—C10A—C11A—C12A	−1.4 (6)	C9B—C10B—C11B—C12B	0.3 (6)
C10A—C11A—C12A—C13A	0.8 (5)	C10B—C11B—C12B—C13B	1.2 (5)
C7A—C8A—C13A—C14A	−0.2 (4)	C11B—C12B—C13B—C14B	178.0 (3)
C9A—C8A—C13A—C14A	178.8 (3)	C11B—C12B—C13B—C8B	−1.8 (4)
C7A—C8A—C13A—C12A	179.0 (3)	C7B—C8B—C13B—C14B	0.6 (4)
C9A—C8A—C13A—C12A	−2.0 (4)	C9B—C8B—C13B—C14B	−178.9 (3)
C11A—C12A—C13A—C14A	−179.9 (3)	C7B—C8B—C13B—C12B	−179.6 (3)
C11A—C12A—C13A—C8A	0.9 (4)	C9B—C8B—C13B—C12B	0.9 (4)
C2A—C1A—C14A—C13A	178.4 (2)	C12B—C13B—C14B—C1B	179.9 (3)
C6A—C1A—C14A—C13A	−0.8 (4)	C8B—C13B—C14B—C1B	−0.3 (4)
C2A—C1A—C14A—C15A	−1.6 (4)	C12B—C13B—C14B—C15B	2.0 (4)
C6A—C1A—C14A—C15A	179.2 (2)	C8B—C13B—C14B—C15B	−178.2 (2)
C8A—C13A—C14A—C1A	0.9 (4)	C2B—C1B—C14B—C13B	−178.9 (3)
C12A—C13A—C14A—C1A	−178.3 (2)	C6B—C1B—C14B—C13B	0.0 (4)
C8A—C13A—C14A—C15A	−179.1 (2)	C2B—C1B—C14B—C15B	−1.0 (4)
C12A—C13A—C14A—C15A	1.7 (4)	C6B—C1B—C14B—C15B	177.9 (2)
C1A—C14A—C15A—O1A	−95.2 (4)	C13B—C14B—C15B—O1B	104.3 (3)
C13A—C14A—C15A—O1A	84.8 (4)	C1B—C14B—C15B—O1B	−73.6 (4)
C1A—C14A—C15A—C16A	84.8 (3)	C13B—C14B—C15B—C16B	−75.7 (4)
C13A—C14A—C15A—C16A	−95.2 (3)	C1B—C14B—C15B—C16B	106.3 (3)
O1A—C15A—C16A—C17A	4.4 (5)	O1B—C15B—C16B—C17B	−172.6 (3)
C14A—C15A—C16A—C17A	−175.6 (3)	C14B—C15B—C16B—C17B	7.4 (5)
C15A—C16A—C17A—C18A	179.7 (3)	C15B—C16B—C17B—C18B	173.5 (3)
C16A—C17A—C18A—C23A	7.6 (4)	C16B—C17B—C18B—C23B	−166.7 (3)
C16A—C17A—C18A—C19A	−171.2 (3)	C16B—C17B—C18B—C19B	11.4 (5)
C23A—C18A—C19A—C20A	−1.6 (4)	C23B—C18B—C19B—C20B	0.1 (4)
C17A—C18A—C19A—C20A	177.4 (3)	C17B—C18B—C19B—C20B	−178.0 (3)
C18A—C19A—C20A—C21A	1.2 (4)	C18B—C19B—C20B—C21B	−1.5 (5)
C19A—C20A—C21A—C22A	−0.1 (4)	C19B—C20B—C21B—C22B	1.8 (4)
C19A—C20A—C21A—N1A	−178.7 (3)	C19B—C20B—C21B—N1B	−177.8 (3)
C35A—N1A—C21A—C22A	57.7 (4)	C24B—N1B—C21B—C22B	−133.4 (3)
C24A—N1A—C21A—C22A	−114.5 (3)	C35B—N1B—C21B—C22B	49.8 (4)
C35A—N1A—C21A—C20A	−123.8 (3)	C24B—N1B—C21B—C20B	46.2 (4)
C24A—N1A—C21A—C20A	64.1 (4)	C35B—N1B—C21B—C20B	−130.6 (3)
C20A—C21A—C22A—C23A	−0.6 (4)	C20B—C21B—C22B—C23B	−0.9 (4)
N1A—C21A—C22A—C23A	178.0 (2)	N1B—C21B—C22B—C23B	178.7 (3)
C21A—C22A—C23A—C18A	0.2 (4)	C21B—C22B—C23B—C18B	−0.5 (4)
C19A—C18A—C23A—C22A	0.9 (4)	C19B—C18B—C23B—C22B	0.8 (4)
C17A—C18A—C23A—C22A	−178.0 (2)	C17B—C18B—C23B—C22B	179.1 (3)
C35A—N1A—C24A—C25A	−178.9 (3)	C35B—N1B—C24B—C25B	−176.2 (3)
C21A—N1A—C24A—C25A	−5.6 (5)	C21B—N1B—C24B—C25B	6.4 (4)
C35A—N1A—C24A—C29A	0.7 (3)	C35B—N1B—C24B—C29B	0.3 (3)
C21A—N1A—C24A—C29A	174.0 (2)	C21B—N1B—C24B—C29B	−177.1 (2)
C29A—C24A—C25A—C26A	1.5 (4)	C29B—C24B—C25B—C26B	2.1 (4)
N1A—C24A—C25A—C26A	−178.9 (3)	N1B—C24B—C25B—C26B	178.2 (3)
C24A—C25A—C26A—C27A	−0.3 (5)	C24B—C25B—C26B—C27B	−0.7 (4)
C25A—C26A—C27A—C28A	−0.9 (5)	C25B—C26B—C27B—C28B	−0.9 (5)
C26A—C27A—C28A—C29A	0.9 (5)	C26B—C27B—C28B—C29B	1.1 (5)
C25A—C24A—C29A—C28A	−1.5 (4)	C27B—C28B—C29B—C24B	0.4 (4)
N1A—C24A—C29A—C28A	178.8 (2)	C27B—C28B—C29B—C30B	−178.4 (3)
C25A—C24A—C29A—C30A	178.4 (3)	C25B—C24B—C29B—C28B	−2.0 (4)
N1A—C24A—C29A—C30A	−1.3 (3)	N1B—C24B—C29B—C28B	−178.8 (2)
C27A—C28A—C29A—C24A	0.2 (4)	C25B—C24B—C29B—C30B	177.0 (2)
C27A—C28A—C29A—C30A	−179.6 (3)	N1B—C24B—C29B—C30B	0.2 (3)
C24A—C29A—C30A—C31A	179.3 (3)	C28B—C29B—C30B—C31B	−3.8 (6)
C28A—C29A—C30A—C31A	−0.9 (6)	C24B—C29B—C30B—C31B	177.3 (3)
C24A—C29A—C30A—C35A	1.4 (3)	C28B—C29B—C30B—C35B	178.3 (3)
C28A—C29A—C30A—C35A	−178.8 (3)	C24B—C29B—C30B—C35B	−0.6 (3)
C35A—C30A—C31A—C32A	0.3 (4)	C35B—C30B—C31B—C32B	−0.4 (5)
C29A—C30A—C31A—C32A	−177.4 (3)	C29B—C30B—C31B—C32B	−178.1 (3)
C30A—C31A—C32A—C33A	1.5 (5)	C30B—C31B—C32B—C33B	−0.5 (5)
C31A—C32A—C33A—C34A	−1.2 (5)	C31B—C32B—C33B—C34B	0.9 (5)
C32A—C33A—C34A—C35A	−1.0 (5)	C32B—C33B—C34B—C35B	−0.3 (5)
C33A—C34A—C35A—N1A	178.8 (3)	C33B—C34B—C35B—C30B	−0.6 (4)
C33A—C34A—C35A—C30A	2.9 (4)	C33B—C34B—C35B—N1B	177.6 (3)
C24A—N1A—C35A—C34A	−176.3 (3)	C31B—C30B—C35B—C34B	1.0 (4)
C21A—N1A—C35A—C34A	10.5 (5)	C29B—C30B—C35B—C34B	179.2 (2)
C24A—N1A—C35A—C30A	0.1 (3)	C31B—C30B—C35B—N1B	−177.5 (2)
C21A—N1A—C35A—C30A	−173.1 (2)	C29B—C30B—C35B—N1B	0.7 (3)
C31A—C30A—C35A—C34A	−2.6 (4)	C24B—N1B—C35B—C34B	−179.0 (3)
C29A—C30A—C35A—C34A	175.7 (3)	C21B—N1B—C35B—C34B	−1.7 (4)
C31A—C30A—C35A—N1A	−179.2 (2)	C24B—N1B—C35B—C30B	−0.6 (3)
C29A—C30A—C35A—N1A	−0.9 (3)	C21B—N1B—C35B—C30B	176.6 (2)

### (E)-1-(Anthracen-9-yl)-3-[4-(9H-carbazol-9-yl)phenyl]prop-2-en-1-one (mo_DA21e_0m) Hydrogen-bond geometry (Å, º)

**Table:**

*D*—H···*A*	*D*—H	H···*A*	*D*···*A*	*D*—H···*A*
C12*B*—H12*B*···O1*B*^i^	0.93	2.51	3.266 (4)	138
C5*B*—H5*BA*···*Cg*6^ii^	0.93	2.79	3.585 (4)	144
C27—H27*B*···*Cg*7	0.93	2.85	3.577 (4)	136
C28—H28*B*···*Cg*8	0.93	2.70	3.382 (4)	130
C11—H11*A*···*Cg*9^iii^	0.93	2.85	3.742 (4)	161
C7—H7*BA*···*Cg*10^ii^	0.93	2.90	3.704 (3)	145

Symmetry codes: (i) *x*, −*y*+1/2, *z*+1/2; (ii) −*x*+1, −*y*, −*z*; (iii) *x*, *y*, *z*+1.
