# Supplementary material for: Crystal structure and theoretical studies of two π-conjugated fused-ring chalcones: (E)-1-(anthra­cen-9-yl)-3-(9-ethyl-9H-carbazol-3-yl)prop-2-en-1-one and (E)-1-(anthracen-9-yl)-3-[4-(9H-carbazol-9-yl)phen­yl]prop-2-en-1-one
Source: Acta Crystallogr E Crystallogr Commun. 2018 Aug 21;74(Pt 9):1302–8. doi: 10.1107/S2056989018011131 (PMC6127721; doi:10.1107/S2056989018011131)
Supplement: Supplementary file 5 [file e-74-01302-sup5.pdf]

STRUCTURE REPORTS

Volume 74 (2018)

Supporting information for article:

Crystal structure and theoretical studies of the π-conjugated fused-ring chalcones: (*E*)-1-(anthracen-9-yl)-3-(9-ethyl-9*H*-carbazol-3-yl)prop-2-en-1-one and (*E*)-1-(anthracen-9-yl)-3-[4-(9*H*-carbazol-9-yl)phenyl]prop-2-en-1-one

Dian Alwani Zainuri, Ibrahim Abdul Razak and Suhana Arshad

Crystal Structure and Theoretical Studies of Linear Fused Ring Chalcone : (*E*)-1-(anthracen-9-yl)-3-(9-ethyl-9*H*-carbazol-3-yl)prop-2-en-1-one and (*E*)-3-(4-(9*H*-carbazol-9-yl)phenyl)-1-(anthracen-9-yl)prop-2-en-1-one

Table S1 A Comparison Selected Single Crystal X-Ray data and DFT Geometry Optimized Data for Compounds (**I**) and (**II**).

	**Compound I**		**Compound II**		
**Bonds**	**X-ray (Experimental) (Å^0)**	**DFT* (Theoretical) (Å^0)**	**X-ray (Experimental) (Å^0)**		**DFT* (Theoretical) (Å^0)**
			**A**	**B**	
C15-O1	1.22 (2)	1.22	1.21 (3)	1.22 (3)	1.22
C1-C14	1.41 (2)	1.41	1.40 (4)	1.40 (4)	1.41
C1-C2	1.42 (3)	1.43	1.42 (4)	1.42 (4)	1.43
C2-C3	1.35 (3)	1.37	1.36 (5)	1.36 (5)	1.37
C3-C4	1.41 (3)	1.42	1.40 (5)	1.40 (5)	1.42
C4-C5	1.35 (3)	1.37	1.34 (5)	1.34 (5)	1.37
C5-C6	1.42 (3)	1.43	1.42 (4)	1.42 (4)	1.43
C6-C7	1.39 (3)	1.40	1.39 (4)	1.39 (4)	1.40
C7-C8	1.39 (2)	1.40	1.39 (4)	1.38 (4)	1.40
C8-C9	1.42 (3)	1.43	1.44 (4)	1.42 (4)	1.43
C9-C10	1.35 (3)	1.37	1.35 (5)	1.35 (5)	1.37
C10-C11	1.41 (3)	1.42	1.40 (5)	1.40 (5)	1.42
C11-C12	1.35 (3)	1.37	1.35 (4)	1.35 (4)	1.37
C12-C13	1.43 (2)	1.43	1.43 (4)	1.42 (4)	1.43
C13-C14	1.40 (2)	1.41	1.40 (4)	1.40 (4)	1.41
C14-C15	1.51 (2)	1.52	1.50 (4)	1.51 (4)	1.52
C15-C16	1.46 (3)	1.47	1.46 (4)	1.46 (4)	1.47
C16-C17	1.33 (2)	1.35	1.32 (3)	1.32 (4)	1.35
C17-C18	1.46 (2)	1.46	1.460 (3)	1.46 (4)	1.46
C14—C15—C16	119.00 (16)	119.34	118.9 (2)	120.0 (3)	119.37
O1—C15—C14	119.96 (17)	119.78	119.9 (2)	119.9 (3)	120.24

O1—C15—C16	121.04 (18)	120.89	121.2 (3)	120.1 (3)	120.39
C15—C16—C17	124.03 (17)	124.09	122.4 (3)	124.0 (3)	124.15
C16—C17—C18	126.96 (17)	128.01	129.0 (3)	128.0 (3)	127.48
C1—C14—C15—O1	88.2 (2)	94.36	−95.2 (4)	−73.6 (4)	-94.78
C13—C14—C15—O1	−90.0 (2)	-83.52	84.8 (4)	104.3 (3)	83.31
C1—C14—C15—C16	−92.6 (2)	-85.84	84.8 (3)	106.3 (3)	85.63
C13—C14—C15—C16	89.2 (2)	96.28	−95.2 (3)	−75.7 (4)	-96.28
O1—C15—C16—C17	173.6 (2)	177.91	4.4 (5)	−172.6 (3)	-177.97
C14—C15—C16—C17	−5.6 (3)	-1.89	−175.6 (3)	7.4 (5)	1.61
C15—C16—C17—C18	−175.80 (18)	179.97	179.7 (3)	173.5 (3)	179.69
C16—C17—C18—C19	−16.4 (3)	-1.38	−171.2 (3)	11.4 (5)	-1.70

* B3LPY/6-311++G(d,p)
